# Antioxidant and Skin-Whitening Efficacy of a Novel Decapeptide (DP, KGYSSYICDK) Derived from Fish By-Products

**DOI:** 10.3390/md22080374

**Published:** 2024-08-20

**Authors:** Sung-Gyu Lee, Jin-Woo Hwang, Hyun Kang

**Affiliations:** 1 Department of Medical Laboratory Science, College of Health Science, Dankook University, Cheonan-si 31116, Chungcheongnam-do, Republic of Korea; croucard@naver.com; 2 Marine Bio-Food and Drug Convergence Technology Center, Dankook University, Cheonan-si 31116, Chungcheongnam-do, Republic of Korea

**Keywords:** peptide, fish by product, skin whitening, tyrosinase, B16F10

## Abstract

The skin is vulnerable to damage from ultraviolet rays and oxidative stress, which can lead to aging and pigmentation issues. This study investigates the antioxidant and whitening efficacy of a decapeptide (DP, KGYSSYICDK) derived from marine fish by-products and evaluates its potential as a new skin-whitening agent. DP demonstrated high antioxidant activity, showing comparable or superior performance to Vitamin C (Vit. C) in ferric reducing antioxidant power (FRAP) and 2,2′-azino-bis(3-ethylbenzothiazoline-6-sulfonic acid) (ABTS) radical scavenging assays. In hydrogen peroxide (H_2_O_2_)-treated HaCaT cells, DP increased cell viability and reduced reactive oxygen species (ROS) generation. Furthermore, DP inhibited tyrosinase activity and decreased melanin production in α-melanocyte stimulating hormone (α-MSH)-induced B16F10 melanoma cells in a dose-dependent manner. Reverse transcription polymerase chain reaction (RT-PCR) analysis revealed that DP reduces the mRNA expression of *MITF*, *tyrosinase*, and *MC1R*, thus suppressing melanin production. DP exhibits strong binding interactions with multiple amino acid residues of tyrosinase, indicating potent inhibitory effects on the enzyme. These results suggest that DP possesses significant antioxidant and whitening properties, highlighting its potential as a skin-whitening agent. Future research should focus on optimizing DP’s structure and exploring structure–activity relationships.

## 1. Introduction

The skin is directly exposed to various oxidative stresses due to environmental factors. Prolonged exposure to ultraviolet rays can lead to the excessive generation of reactive oxygen species (ROS), damaging the antioxidant defense system within cells and accelerating the expression of genes related to oxidative stress [1,2], pigmentation disorders, photoaging, skin cancer, cataracts, immune system impairment, and cancer induction [3,4]. To counteract these effects, recent research has focused on extracting useful components from natural sources with low toxicity for developing food, medical materials, and anti-aging or whitening cosmetic ingredients.

Melanin is a stable polymeric pigment found in both plants and animals. It is synthesized in melanocytes through a complex, multi-step process involving the enzyme tyrosinase, which catalyzes the conversion of l-Tyrosine to 3,4-dihydroxyphenylalanine (DOPA), and subsequently to dopaquinone [5]. Melanin production is stimulated by factors like ultraviolet rays and oxidative stress, leading to the release of α-melanocyte stimulating hormone (α-MSH), which triggers melanogenesis by activating signaling pathways in melanocytes [6]. α-MSH is produced in various skin cells such as melanocytes and keratinocytes, as well as in the intermediate lobe of the pituitary gland and the hypothalamus. It binds to the melanocortin receptor (MC1R), which is a membrane receptor specifically expressed in melanocytes. This binding activates adenylyl cyclase, amplifies intracellular cAMP signaling, and induces the activation of protein kinase A (PKA). Through the activation of cAMP response element-binding protein (CREB), it increases the expression of the microphthalmia-associated transcription factor (MITF), which is a basic helix–loop–helix leucine zipper (b-HLH-Zip) transcription factor in melanocytes [5].

Current skin-whitening strategies primarily involve either reducing existing melanin or inhibiting tyrosinase, the enzyme responsible for melanin synthesis [7]. Various chemical agents and plant extracts have been used in skin-whitening products, but issues such as discoloration, odor, and cytotoxicity have limited their application [8]. Therefore, there is a need for effective whitening agents that are free from adverse effects like skin irritation and allergic reactions. Many studies have been conducted to explore various whitening mechanisms [9,10]. 

Marine fish proteins are composed of small peptides and often exist in an inactive form along with the entire protein sequence. Enzymatic hydrolysis is frequently used to isolate short, bioactive peptides from marine organisms and seafood waste [11]. Peptides serve as important active components in various pharmaceutical and cosmeceutical applications [12,13]. Bioactive peptides typically consist of 3–20 amino acid residues and exhibit a range of biological activities [14,15,16]. 

In previous research, we identified a decapeptide (DP, KGYSSYICDK) from fish by-products with free radical scavenging and tyrosinase inhibitory activities [17]. This study aims to evaluate the antioxidant and skin-whitening effects of this novel decapeptide (DP) through in vitro and in silico analyses and to establish its potential as a new skin-whitening agent.

## 2. Results

### 2.1. Structural Characterization of DP

**Figure 1:**
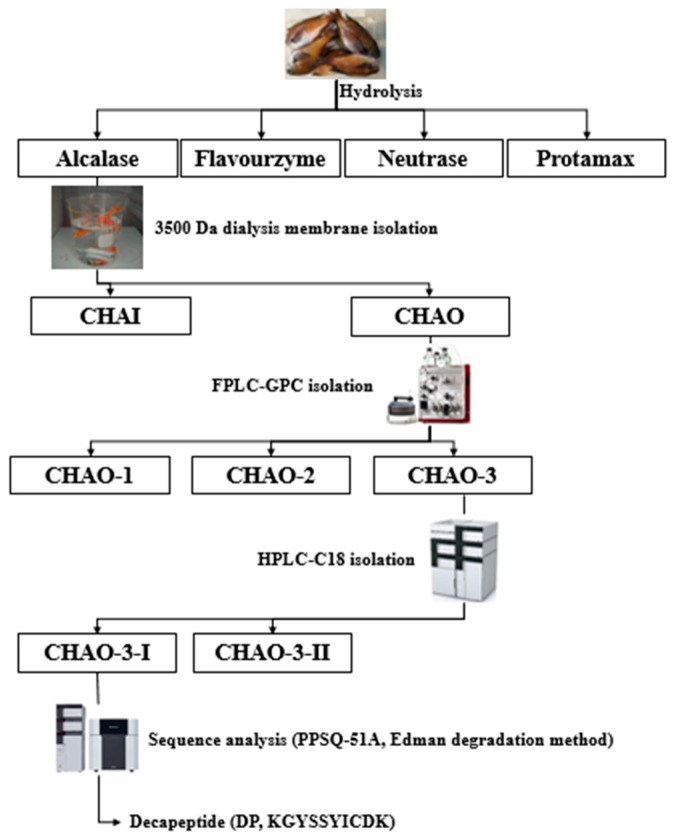
Identification of DP peptide (DP, KGYSSYICDK) from *C. notate* by-product hydrolysate.

**Figure 2:**
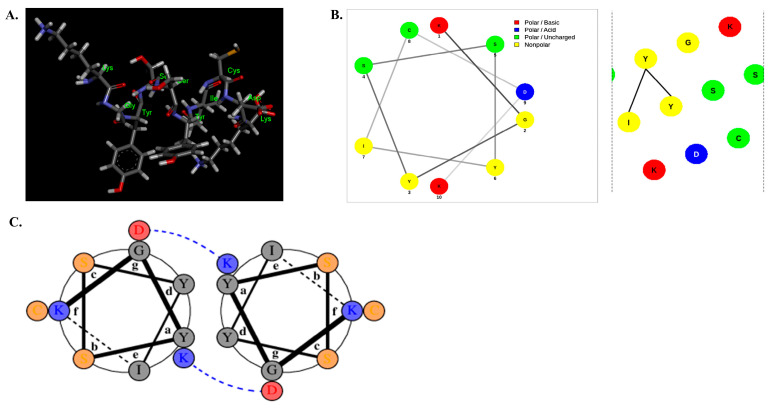
Structural characterization of *C. notate* by-product derived from the DP peptide: (**A**) Chemical structure of the DP peptide. (**B**) An example of a helical wheel diagram illustrated for the DP peptide, showing the net projection (**left panel**) and the wheel projection (**right panel**) for the DP peptide. (**C**) Antiparallel alignment of two helical wheels of the DP peptide.

The process of preparation of *C. notate* by-product hydrolysate, measurement of ABTS radical scavenging activity of the hydrolysate, amino acid sequencing and synthesis, and isolation of the decapeptide by amino acid sequencing and synthesis is shown in Figure 1. Figure 2A shows the 3D virtual structure of the DP peptide. The wheel and net projections have been proposed to represent in two dimensions the tridimensional helical structures and facilitate the observation of their properties, especially in terms of residue polarity and intramolecular bonding (Figure 2B). Net projections are used for the same structures as wheels but provide a different perspective to the visualization of the helixes. At least two chains must be specified as a homodimer (Figure 2C).

These results suggest that DP peptides are likely to adopt α-helix-dominated conformations upon interactions with cell membranes, and the mechanism of action of the peptides could be anticipated via their amphipathic helical properties.

### 2.2. Mass Spectra of DP

**Figure 3:**
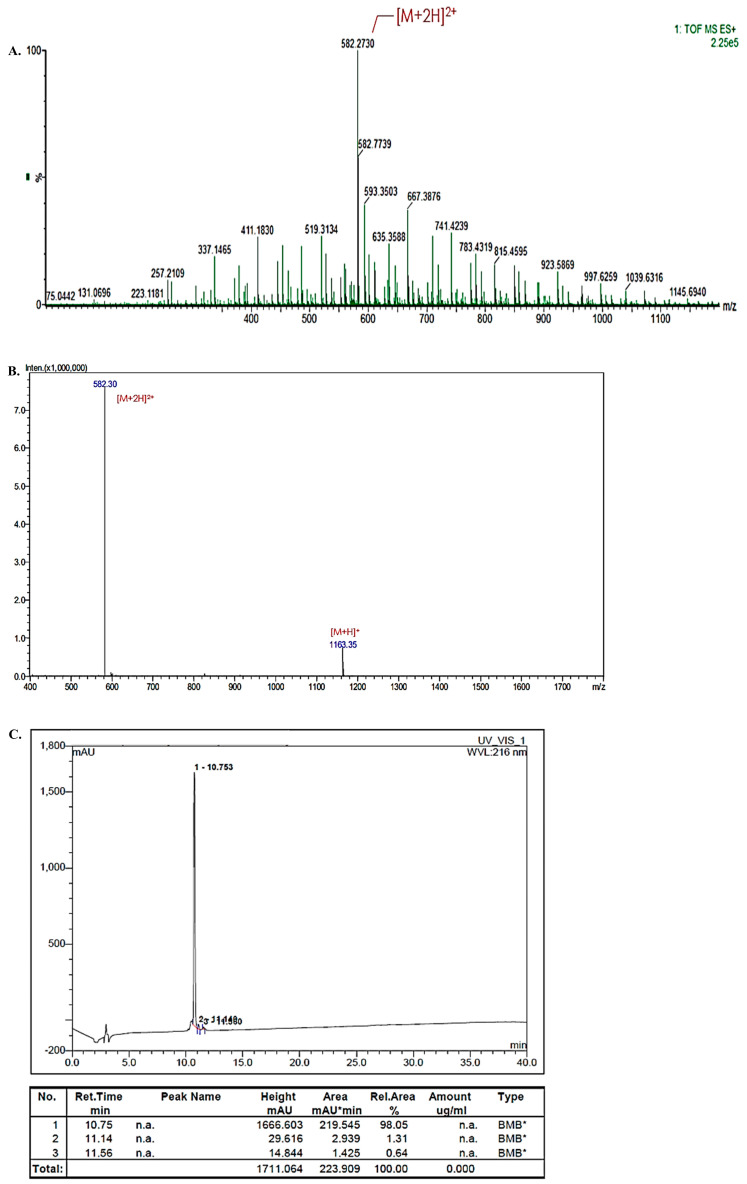
The synthesis process of *C. notate* by-product-derived DP peptide (DP, decapeptide, KGYSSYICDK): (**A**) Mass spectrum of DP analyzed by LC-HRMS. (**B**) Mass spectrum of DP analyzed by LC-MS. (**C**) Purity of DP analyzed by RP-HPLC. BMB*: 6, 4-tert-Butyl-4’-methoxydibenzoylmethane, mAU*min: Milli-Absorbance Units*minute.

Figure 3A shows the result of the mass spectrum of DP analyzed by LC-HRMS. Figure 3B. shows the result of the mass spectrum of DP analyzed by LC-MS. And Figure 3C shows the result of the purity of DP analyzed by RP-HPLC.

### 2.3. Effects of DP in Ferric Reducing Antioxidant Power (FRAP) and 2,2′-Azino-bis(3-ethylbenzothiazoline-6-sulfonic Acid) (ABTS) Radical Scavenging Activity

**Figure 4:**
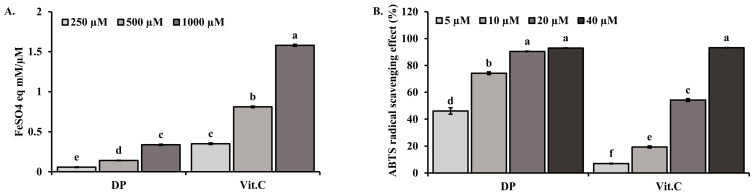
Antioxidant FRAP activity and radical scavenging activity of DP: (**A**) Reducing power of DP and Vit. C FRAP value, analyzed as FeSO_4_ equivalents (FeSO_4_E) in mM/μM of the sample. (**B**) ABTS radical scavenging activities of DP and Vit. C. Data are expressed as the means ± standard deviations (*n* = 3). Different superscripts in a column indicate significant differences at *p* < 0.05 using Duncan’s multiple range test.

To compare the antioxidant activity of DP, FRAP values were measured against FeSO_4_ standards (Figure 4A). DP exhibited dose-dependent FRAP activity, with significant activity at 1000 μM, comparable to Vitamin C (Vit. C, *p* < 0.05). Additionally, DP demonstrated concentration-dependent ABTS radical scavenging activity, with over 90% scavenging at concentrations above 20 μM, significantly outperforming Vit. C (*p* < 0.05) (Figure 4B).

### 2.4. Inhibition of Hydrogen Peroxide (H_2_O_2_)-Induced Cell Damage and Reactive Oxygen Species (ROS) Reduction by DP in HaCaT Cells

**Figure 5:**
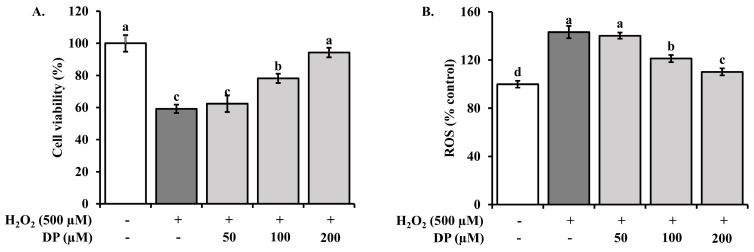
Effect of DP on cell viability and ROS in H_2_O_2_-treated HaCaT human keratinocytes; HaCaT cells were treated with different concentrations of DP (50, 100, 200 μM) and H_2_O_2_ (500 μM) for 24 h: (**A**) The cell viability was assessed by the MTT reduction assay, and the results are expressed as the percentage of surviving cells over the control cells (no addition of DP and H_2_O_2_). (**B**) HaCaT cells were incubated with 25 µM of DCFH-DA for 30 min, followed by measurement of fluorescent DCFH-DA production. Results are expressed as DCFH-DA fluorescence relative to untreated controls. Data are expressed as the means ± standard deviations (*n* = 3). Different superscripts in a column indicate significant differences at *p* < 0.05 using Duncan’s multiple range test.

To assess DP’s protective effects against H_2_O_2_-induced damage, HaCaT cells were treated with 50, 100, and 200 μM of DP and subjected to 3-(4,5-dimethylthiazol-2-yl)-2,5-diphenyltetrazolium bromide (MTT) assays. DP enhanced cell viability in a dose-dependent manner (Figure 5A). ROS levels, measured with Dichloro-dihydro-fluorescein diacetate (DCFH-DA), were significantly reduced by DP treatment (Figure 5B), indicating its potential to mitigate oxidative stress.

### 2.5. Inhibition of Tyrosinase Activity by DP

**Figure 6:**
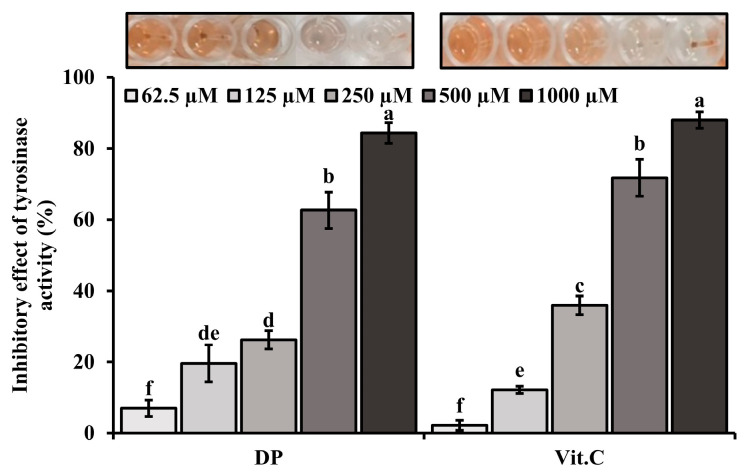
Inhibitory effect of DP on tyrosinase. The tyrosinase activity assay was performed using mushroom tyrosinase. Different superscripts in a column indicate significant differences at *p* < 0.05 using Duncan’s multiple range test.

Tyrosinase, a copper-containing enzyme, plays a crucial role in melanin formation. To effectively inhibit the synthesis of melanin polymers, the tyrosinase inhibitory activity of DP was measured against mushroom-derived tyrosinase (Figure 6). At concentrations of 62.5, 125, 250, 500, and 1000 μM, DP exhibited tyrosinase inhibitory activities of 7.05%, 19.62%, 26.29%, 62.67%, and 84.38%, respectively. When compared to Vit. C, a standard whitening agent, at the same concentration, DP showed similar tyrosinase inhibitory activity, suggesting its potential as a functional material for whitening.

### 2.6. Effect of DP on Melanin Content and Morphology in α-MSH-Induced B16F10 Melanoma Cells

**Figure 7:**
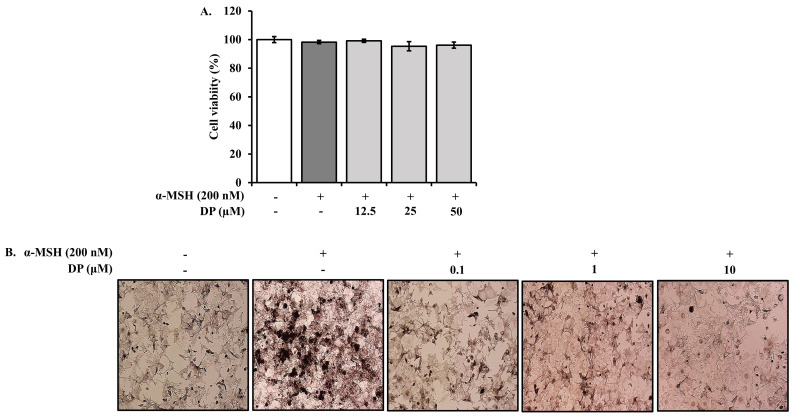

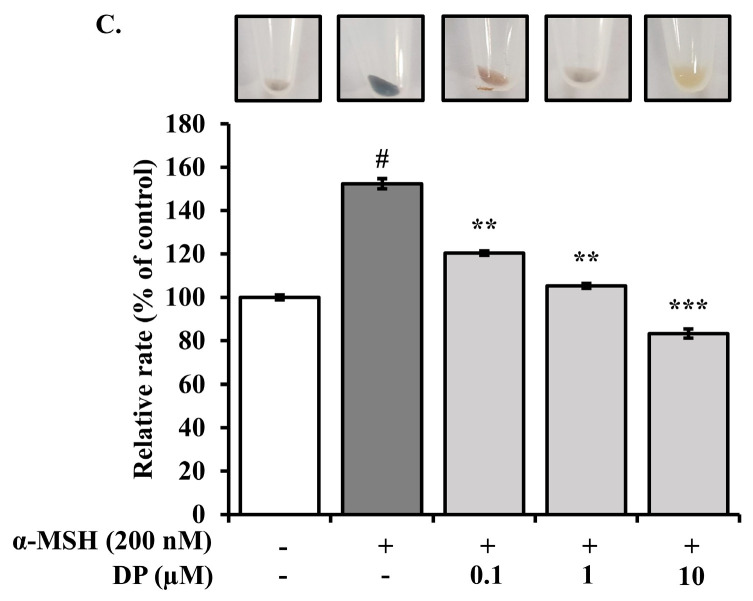
Effect of DP on cell viability and melanin content in α-MSH-induced melanogenesis in B16F10 melanoma cells; B16F10 melanoma cells were treated with different concentrations of DP and α-MSH (200 nM) for 24 h: (**A**) Cytotoxicity of DP in B16F10 melanoma cells (1 × 10^4^ cells/mL) by MTT assay. (**B**) Changes in morphology (200× magnification) and (**C**) melanin content due to DP on melanogenesis in B16F10 melanoma cells. All data are expressed as the mean ± SD of the experiment. # *p* < 0.05 compared to the control group; ** *p* < 0.01 and *** *p* < 0.001 compared to the α-MSH control group.

Before measuring the melanin content in B16F10 melanoma cells, the cytotoxicity of DP was assessed at concentrations of 12.5, 50, and 100 μM. No cytotoxicity was observed at any of these concentrations after 24 h (Figure 7A). For further analysis, B16F10 melanoma cells were treated with DP at concentrations of 0.1, 1, and 10 μM, along with α-MSH (200 nM), and incubated for 24 h. The melanin production was then measured. Morphological observations confirmed that melanin formation was inhibited starting from the 0.1 μM concentration of DP (Figure 7B). The results of the melanin production assay showed that the melanin content increased by 152.36% in the α-MSH treated group compared to the control group. In contrast, melanin production decreased to 120.32%, 105.24%, and 83.21% at 0.1, 1, and 10 μM concentrations of DP, respectively, compared to the α-MSH treated group (Figure 7C).

### 2.7. Inhibition of MITF, Tyrosinase, and MC1R mRNA Expression by DP

**Figure 8:**
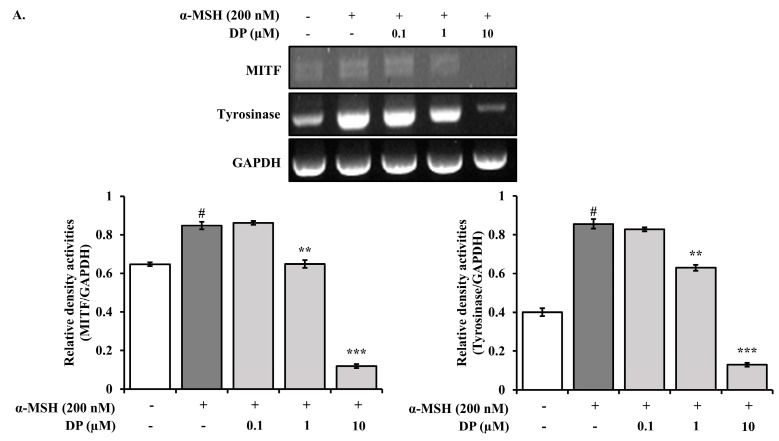

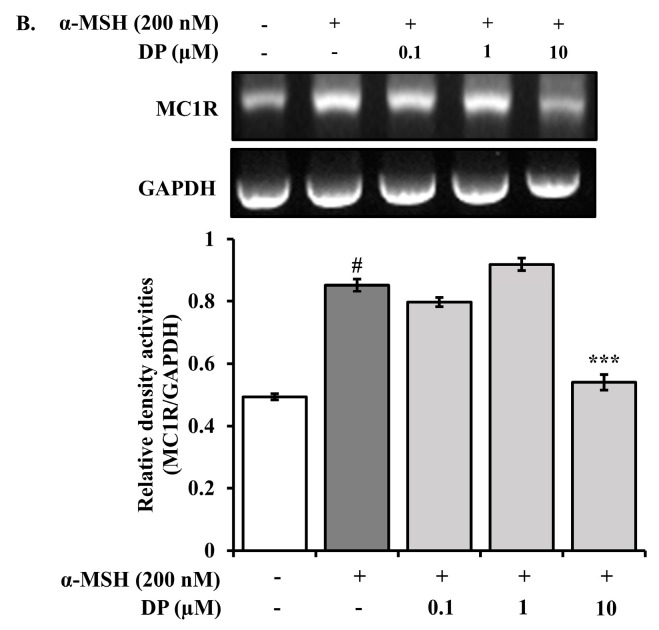
Effects of DP on mRNA expression of *MITF*, *tyrosinase*, *MC1R* in B16F10 cells stimulated with α-MSH. B16F10 cells were co-treated with DP and 200 nM α-MSH. After treatment, the mRNA expression of (**A**) *MITF*, *tyrosinase*, and (**B**) *MC1R* was measured and normalized to GAPDH expression. All data are expressed as the mean ± SD of the experiment. # *p* < 0.05 compared to the control group; ** *p* < 0.01 and *** *p* < 0.001 compared to the α-MSH control group.

To evaluate whether DP affects the expression of melanin-related genes such as *MITF*, *tyrosinase*, and *MC1R*, Reverse transcription polymerase chain reaction (RT-PCR) was used to investigate gene expression in α-MSH-induced B16F10 cells. Since previous experiments indicated that DP at concentrations of 0.1, 1, and 10 μM significantly affected melanin synthesis, gene expression was also assessed at these concentrations. As shown in Figure 8, treatment with DP at concentrations of 0.1, 1, and 10 μM resulted in a dose-dependent and significant decrease in the mRNA levels of *MITF*, *tyrosinase*, and *MC1R*. These results suggest that DP downregulates the expression of *MITF*, *tyrosinase*, and *MC1R* mRNA, thereby influencing melanin production in α-MSH-stimulated B16F10 cells.

### 2.8. Molecular Docking Model of DP with Tyrosinase Protein

**Figure 9:**
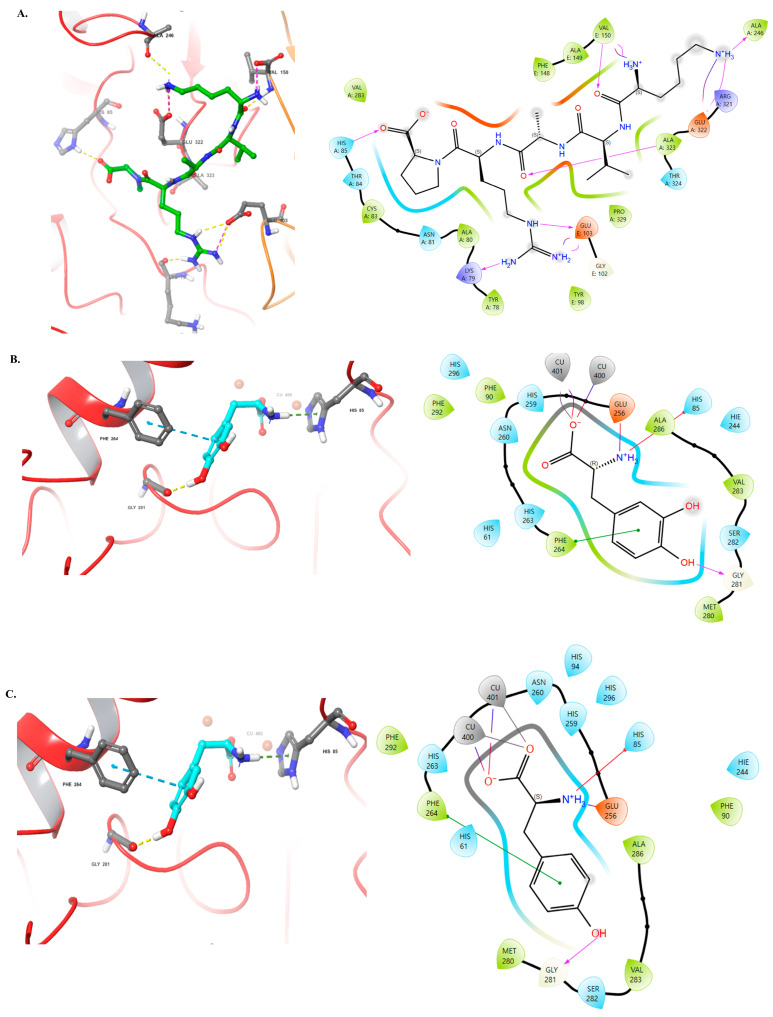

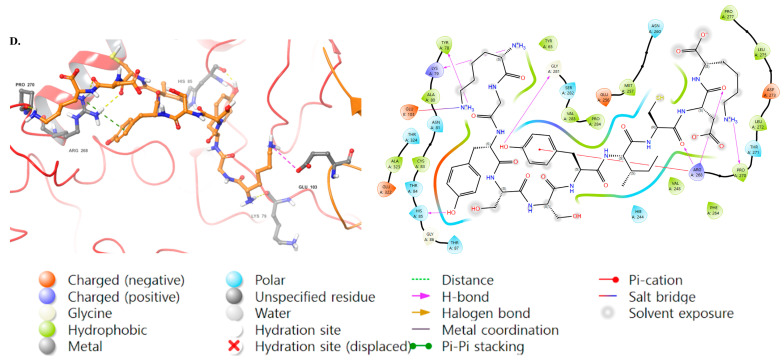
The molecular docking of mushroom tyrosinase(2Y9X) with KVARP (**A**, **right panel**), L-TYROSINE (**B**, **right panel**), L-DOPA (**C**, **right panel**), and KGYSSYICDK (**D**, **right panel**) peptide, respectively. Two-dimensional (2D) diagrams of predicted interactions between KVARP (**A**, **left panel**), L-TYROSINE (**B**, **left panel**), L-DOPA (**C**, **left panel**), and KGYSSYICDK (**D**, **left panel**) peptide with mushroom tyrosinase, only displayed interacting residues. The ligand is shown in the stick model (yellow stands for carbon; blue stands for nitrogen; red stands for oxygen; green stands for halogen).

The 3D structure of mushroom tyrosinase, and the bioactive effects of KVARP (Figure 9A), L-TYROSINE (Figure 9B), L-DOPA (Figure 9C), and KGYSSYICDK (Figure 9D) peptide were concatenated with Schrödinger Suite software. Water molecules in the protein-crystal structure must be removed before the docking procedure. The molecular docking results about mushroom tyrosinase KVARP, L-TYROSINE, L-DOPA, and KGYSSYICDK peptide, respectively, were showed in best interaction poses with −8.64 kcal/mol of docking score and −19.35 kcal/mol of MMGBSA score (Figure 9D). Current commercially used KVARP is −6.66 kcal/mol of docking score and −18.40 kcal/mol of MMGBSA score (Figure 9A), while L-tyrosine and L-DOPA were −9.01 and −8.39 kcal/mol of docking score and −14.60 and −15.08 kcal/mol of MMGBSA score (Figure 9B,C). KGYSSYICDK peptide has numerous interactions with the amino acid residue of human tyrosinase and is similar to that of mushroom tyrosinase (Figure 9D). 

The structure of KVARP, L-TYROSINE, and L-DOPA is shown in yellow, with the main amino acid sequence identified as HIS85, VAL150, GLU103, GLU322, ALA323, ALA246, and LYS79; HIS85, GLY281, PHE264, and CU400; PHE264, GLY 281, GLU256, HIS85, and CU400 (Figure 9A–C, left panel). The structure of KGYSSYICDK (Figure 9D, left panel) identified as LYS79, HIS85, ASP348, GLU103, ARG268, and PRO270. The backbones of TYR78 and GLU103 interact with the NH2 moiety of KGYSSYICDK via hydrogen bonds. The backbone of another GLY281, HIS85, and ARG285 interact with the NH2 moiety of KGYSSYICDK via hydrogen bonding. In addition, the side chains of PRO270 also interact with the NH2 moiety of KGYSSYICDK (Figure 9D, left panel). A two-dimensional representation of the interaction between mushroom tyrosinase (PDB: 2Y9X) and the KVARP, L-TYROSINE, L-DOPA, and KGYSSYICDK peptide active site are shown in Figure 9A–D, right panel.

KVARP, L-TYROSINE, L-DOPA, and KGYSSYICDK had 11, 6, 6, 6, and 14 hydrophobic interactions (lime green), and the polar interaction (light blue) was 4, 8, 9, and 10. The GLY281 interaction (white color) was found in L-TYROSINE and KGYSSYICDK. Interestingly, a negative-charged interaction with GLU256 (Orange color) was found in L-TYROSINE and L-DOPA, and a positive-charged interaction (violet color) was found in KVARP and KGYSSYICDK (Figure 9A–D, right panel).

## 3. Discussion

Marine fish are primarily used as a food source for human consumption, leading to the production of fish meat in various fish processing industries. However, these industries generate large amounts of waste, including heads, tails, and bones, which exacerbates environmental pollution. To address this issue, by-products generated in the seafood processing industry are utilized to extract bioactive compounds beneficial to human health. This process helps reduce pollution and also enhances the value of fish processing by-products [18,19,20]. Fish processing waste contains a significant amount of useful protein, which serves as a source for bioactive peptide extraction [21,22,23]. Peptides have been extensively studied as active ingredients in cosmetics due to their high biocompatibility and protein-mimicking activities [24]. In recent studies, fish-derived peptides have shown promising applications in various fields, including cosmetics, due to their antioxidant and anti-inflammatory properties [25,26]. Furthermore, recent research highlights the potential of fish by-products in producing peptides with significant bioactivities, such as antioxidant, anti-inflammatory, and anti-melanogenic effects [27]. In this study, we evaluated the antioxidant activity of a major bioactive peptide, decapeptide (DP, KGYSSYICDK), found in fish by-products from the Chungcheong West Coast region of the Republic of Korea in HaCaT cells and investigated its anti-melanogenic effects in mouse melanoma (B16F10) cells. Additionally, we conducted in silico analysis with tyrosinase protein to explore its potential as a new skin-whitening agent.

Physiologically beneficial compounds cannot be assessed for antioxidant activity using a single method. Therefore, to investigate and understand these possible mechanisms, we performed FRAP activity and ABTS radical scavenging assays to determine the antioxidant activity of decapeptide. Since antioxidant capacity is strongly related to reducing ability, FRAP analysis is used as a method to measure the antioxidant activity of various compounds [28]. Decapeptide demonstrated a concentration-dependent FRAP activity (Figure 4A). The ABTS free radical scavenging is one of the most widely used spectrophotometric methods to confirm the antioxidant capacity of compounds. Decapeptide’s ABTS free radical scavenging activity was concentration-dependent and continuously improved with increasing sample concentration (Figure 4B). This study demonstrates that decapeptide can scavenge free radicals through a single-electron transfer reaction mechanism (ABTS assay) [29].

H_2_O_2_ is a commonly used oxidant for establishing oxidative stress models, as it can induce excessive ROS production within cells, leading to an imbalance between oxidative and antioxidative levels [30]. In this study, decapeptide treatment improved the survival rate of HaCaT cells exposed to H_2_O_2_ (Figure 5A) and significantly reduced intracellular ROS levels (Figure 5B). This suggests that decapeptide enhances the antioxidant activity of HaCaT cells and can alleviate cell damage induced by H_2_O_2_.

Skin pigmentation primarily occurs in melanocytes of the basal layer of the skin, which are stimulated by ultraviolet (UV) radiation. Stimulated keratinocytes secrete α-MSH [31]. Consequently, UV exposure induces melanin production, leading to hyperpigmentation [32]. The development of whitening agents has primarily focused on inhibiting melanin production in melanocytes by regulating melanin-related factors [33,34,35]. Recently, research has also explored other targets for depigmentation, including the movement, transfer, and degradation of melanosomes, with several substances identified [36,37,38]. Since pigmentation involves various mechanisms, combining treatments that target different aspects may be an effective approach.

Tyrosinase plays a crucial role in melanin biosynthesis [39]. Therefore, the mechanism supporting the efficacy of skin-whitening agents involves reducing tyrosinase activity to inhibit melanin production [40]. The expression of the tyrosinase gene is known to be regulated by MITF [41]. The MC1R, which recognizes α-MSH from external stimuli, activates the CREB signaling pathway. The first step in this process is the conversion of ATP to cAMP. During this process, higher molecular components, including PKA and CREB, are phosphorylated and subsequently transcribe the lower molecular component, MITF [42]. In this study, the anti-melanogenic activity of decapeptide was found to be mediated by inhibiting tyrosinase activity and α-MSH-induced melanin synthesis in B16F10 melanoma cells without inducing cytotoxicity (Figure 7). This anti-melanogenic activity of decapeptide appears to be mediated by the downregulation of mRNA expression of *tyrosinase*, *MITF*, and *MC1R* induced by α-MSH (Figure 8).

The main influence factors of the peptide’s biological activity are the sequence and position of the amino acid [43]. Therefore, molecular docking plays a common and important role in the area of structural molecular biology. There are numerous successful examples obtained with the help of this [44]. Experimental identification of anti-tyrosinases is expensive and time-consuming. Therefore, computational methods provided a preliminary solution to this hypothesis, allowing researchers to use molecular docking to investigate the effect of compounds in the extract on tyrosinase binding and predict and report on their anti-tyrosinase ability. Many studies have used machine learning (ML) methods to create and use a variety of peptide prediction tools, including anti-cancer, anti-inflammatory, anti-biofilm, antibacterial, and cell penetration. This study also utilized the following research. These tools were developed by extracting characteristics such as amino acid composition (AAC), dipeptide composition (DPC), and biochemical composition (BCP) from amino acid sequences. 

A molecular docking study was also conducted to examine the binding conformations of all the synthesized compounds within the catalytic pocket of the enzyme tyrosinase. The docked ligand–protein complexes were investigated based on docking and MMGBSA score. The docking scores had little fluctuations, and the comparison depicted no significant energy difference among all docked molecules due to the similar basic skeleton of the ligands. Therefore, the majority of the ligands showed efficient docking energy values. From docking results, the most active compound KGYSSYICDK was visualized to determine its interactions in the catalytic site of the protein tyrosinase. The interaction energy of the KGYSSYICDK inhibitors was −8.64 kcal/mol of docking score and −19.35 kcal/mol of MMGBSA score, respectively, which were superior to the positive control −6.66 kcal/mol of docking score and −18.40 kcal/mol of MMGBSA score. 

Four strong hydrogen bond contacts were observed. The orthohydroxyl phenolic moiety picks up a hydrogen bond (2.03 Å) interaction with the side chain carbonyl of ARG 268, and this phenolic ring is further stabilized by π–π stacking with side chain HIS85. The keto carbonyl oxygen from the tail moiety of this compound interacts through another hydrogen bond with neighboring PRO270 (1.98 Å), predicting a competitive type of inhibition for KGYSSYICDK. Interestingly, both L-TYROSINE and L-DOPA, PHE264, His 85, His 263, and GLY281 (Cu 401) are conserved residues in the core region. KVARP, L-TYROSINE, L-DOPA, and KGYSSYICDK all bind to HIS85, revealing that this residue is a very important amino acid. It seems to be that tyrosinase inhibition traps histidine amino acid coordinated with Cu ions, which played an important role in the activity.

Our experimental results provided a strong basis for the efficiency of our screening and docking strategies. Our study enriched the function study of human tyrosinase, suggested promising drug candidates for the treatment of hyperpigmentation and malignant melanoma, and provided two potential whitening functional agents. Furthermore, the appropriate structure optimization of KGYSSYICDK and structure–activity relationship study needs to be further studied. 

## 4. Materials and Methods

### 4.1. Experimental Materials

The decapeptide (DP, KGYSSYICDK) used in this study was synthesized by A&Pep Co. (Osong, Republic of Korea). All reagents for antioxidant and whitening assays were purchased from Sigma-Aldrich (St. Louis, MO, USA). For cell culture, fetal bovine serum (FBS), penicillin, and Dulbecco’s Modified Eagle’s Medium (DMEM) were obtained from Gibco BRL Co. (Grand Island, NY, USA).

### 4.2. Peptide Isolation from Hydrolysate of Chromis Notate By-Product

In this study, the heads of *C. notate* by-products were collected from the distribution center of Jeju Island FPC (Fisheries Processing & Marketing Center) in Republic of Korea, and used as hydrolysate preparation samples [17]. The collected *C. notate* heads were freeze-dried in a freeze-dryer (Freeze drying, Vision, Daejeon, Republic of Korea), powdered, and used as hydrolysate samples. One gram of powdered *C. notate* head was added to 50 mL of distilled water with pH 7.0, 20 mg (μL) of hydrolyzing enzyme, and 40 mM of Na_2_SO_3_, and hydrolyzed in a shaking incubator at 50 °C for 8 h. The enzymatic reaction was stopped by centrifugation at 100 °C. The hydrolysate was filtered using Whatman No. 41 filter paper (Whatman Ltd., Maidstone, Kent, UK), freeze-dried in a lyophilizer (Freeze drying, Vision, Daejeon, Republic of Korea), powdered, and stored in a freezer until use (Figure 1).

The ABTS radical scavenging activity of *C. notate* head hydrolysate was determined by an adaptation of the ABTS radical cation decolorization assay of Re et al. [45]. A 7 mM ABTS solution and a 2.45 mM potassium persulfate (Sigma Co., Fukushima, Japan) solution were mixed (*v*/*v*) to a final concentration and allowed to stand in the dark at room temperature (RT) for 24 h. Afterward, distilled water was added to dilute the solution until the absorbance at 732 nm reached 0.70 (±0.02). Then, 990 μL of this diluted ABTS solution was mixed with 10 μL of the sample solution and incubated at RT for 1 min. The absorbance was measured at 732 nm (Figure 1).

To isolate peptides from *C. notate* head hydrolysate, we first used a 3500 Da dialysis membrane to separate the sample in and out of the membrane, then lyophilized it and determined its antioxidant activity by measuring ABTS radical scavenging activity. Fractions with high ABTS radical scavenging activity were separated by FPLC equipped with a GPC (Superdex TM 30 Increase 10/300 GL) column, followed by lyophilization of the fractions. The ABTS radical scavenging activity of the fractions separated using FPLC was measured and the fraction with the best activity was selected. The selected fractions were separated using HPLC equipped with a C18 (ZORBAX SB-C18, 4.6 × 250 mm, 5 μm) column and lyophilized to powder. The ABTS radical scavenging activity of the fractions separated using HPLC was measured to identify the superior fractions (Figure 1).

The amino acid sequence of the final isolated fraction of CHAO-3-1 from *C. notate* by-product hydrolysate was sequenced from the N-terminus using the automated Edman degradation method using a protein sequencer (PPSQ-51A, Shimadzu, Kyoto, Japan) in KAIST (Korea Advanced Institute of Science and Technology, Daejeon, Republic of Korea).

### 4.3. Peptide Synthesis

To identify the molecular weight, analysis was performed by Liquid Chromatography and High-Resolution Mass Spectrometry (LC-HRMS, Waters SYNAPT G2) and it was detected by Ultra Performance Liquid Chromatography (UPLC, Waters Acquity UPLC System, Milford, MA, USA) use for direct injection (injection volume is 5 μL) at the flow rate of 0.25 mL/min, under mobile phase 0.1% formic acid in water/acetonitrile (50:50) direct gradient at isocratic condition. The LC-HRMS analysis conditions included ESI Positive mode, detail conditions are Capillary = +3.1 kv, Sampling cone = 40, Extraction cone = 4.0, Source = 120 °C, Desolvation = 350 °C, Cone gas = 130 L/h, Desolvation gas = 800 L/h and Ms range is 50 to 1200. The analyzed mass-to-charge ratio (*m*/*z*) is shown in the Figure 3A. Mass spectrum was expressed in the form of doubly charged ions.

To identify the molecular weight, Liquid Chromatograph–Mass Spectrometry LC-MS (SHIMADZU LCMS-2020, Kyoto, Japan) analysis was performed, and it was detected by LC-MS at the flow rate of 0.20 mL/min under mobile phase under mobile phase 0.1% formic acid in water/acetonitrile (50:50) using a C_18_ column (particle size 5 µm, 4.6 mm × 250 mm). The LC-MS analysis conditions included ESI Positive mode, Nebulizing gas flow rate = 3 L/min, Curved Desolvation Line (CDL) voltage = −20.0 V, CDL Temp. = 250 °C, Block Heater Temp. 250 °C, Probe Bias = +4.5 kV, and Detector voltage = 1.5 kV. The analyzed mass-to-charge ratio (*m*/*z*) is shown in Figure 3B.

The purity of the synthesized final peptide was analyzed by analytical Reversed-Phase High-Performance Liquid Chromatography (RP-HPLC, Waters 2695, Milford, MA, USA) and UV–Vis Detector. Using a C18 column (particle size 5 µm, 4.6 mm × 250 mm) in condition of measurement wavelength of the detector is 216 nm under the flow rate of 1 mL/min. Acetonitrile (ACN) containing 0.1% trifluoroacetic acid (TFA) and water containing 0.1% TFA were used as the mobile phase solvent of HPLC, and the purity analysis of the peptide was observed by changing the concentration of ACN over time. The purity of DP analyzed according to the measurement value was 98.5% (Figure 3C).

### 4.4. FRAP Measurement

FRAP was measured using the method adapted from Benzie and Strain [46]. The FRAP reagent was prepared by mixing a 300 mM sodium acetate buffer (pH 3.6), a 10 mM 2,4,6-tripyridyl-s-triazine (TPTZ) solution dissolved in 40 mM HCl, and a 20 mM FeCl_3_ solution in a 10:1:1 ratio just before the experiment. A 10 μL sample of each concentration was mixed with 200 μL of FRAP reagent, incubated for 5 min at 37 °C, and then the absorbance was measured at 593 nm using a Microplate Spectrophotometer (xMarkTM, BIO-RAD, Hercules, CA, USA). The FRAP value of the sample was determined by applying the absorbance values to a standard calibration curve prepared with 0–5 mM FeSO_4_·H_2_O, and the results were expressed as FeSO_4_ equivalent mM/μM.

### 4.5. Inhibition of H_2_O_2_-Induced Cell Damage and ROS Removal in HaCaT Cells

#### 4.5.1. HaCaT Cell Culture

The cell line used in the experiment, the HaCaT cell line, a human keratinocyte, was purchased from the Korea Cell Line Bank (KCLB, Seoul, Republic of Korea). HaCaT cells were cultured in a DMEM medium supplemented with 10% FBS and 1% penicillin–streptomycin. The cells were subcultured every 2 to 3 days in an incubator maintained at 37 °C and 5% CO_2_.

#### 4.5.2. Cytotoxicity Test

To assess the cytotoxicity of DP and H_2_O_2_, cells (1 × 10^4^ in a 96-well plate) were cultured at 37 °C and 5% CO_2_ for 24 h. The cultured cells were then treated with DP and H_2_O_2_. After 24 h of treatment, 10 μL of MTT (5 mg/mL) solution was added, and the cells were incubated for 4 h. Following this, the supernatant was removed, and 100 μL of DMSO was added to each well. The absorbance was measured at 550 nm using an ELISA reader.

#### 4.5.3. Measurement of ROS in H_2_O_2_-Induced HaCaT Cells

HaCaT cells were seeded at 1 × 10^4^ cells per well in a 96-well black plate and cultured for 24 h. The cultured cells were treated with DP at concentrations of 50, 100, and 200 μM and then exposed to 500 μM H_2_O_2_ for 24 h to induce ROS generation. After H_2_O_2_ treatment, the cells were stained with 25 μM dichlorodihydro-fluorescein diacetate (DCFH-DA) for 30 min. The fluorescence intensity corresponding to intracellular ROS production was measured using a fluorescent spectrophotometer at an excitation wavelength of 485 nm and an emission wavelength of 530 nm. The results were expressed as ROS production (%) relative to the fluorescence measurement value of the untreated control, which was set to 100.

### 4.6. Measurement of Tyrosinase Inhibitory Activity

The inhibitory activity of tyrosinase, which is involved in the key steps of melanin synthesis, was measured using a colorimetric method modified from Masamoto et al. [47] by detecting the formation of DOPA chrome as a result of the enzyme’s action. As a substrate, a mixture containing 0.2 mL of 5 mM DL-DOPA solution, 0.2 mL of 0.1 M phosphate buffer (pH 6.8), and 0.5 mL of sample solution was prepared. To this mixture, 0.1 mL of mushroom tyrosinase (Sigma Co., 250 unit/mL) was added and incubated at 37 °C for 10 min. After incubation, the absorbance was measured at a wavelength of 475 nm to calculate the tyrosinase inhibitory activity.

### 4.7. Inhibition of Melanin Production in α-MSH Induced B16F10 Melanoma Cells

#### 4.7.1. B16F10 Melanoma Cell Culture

B16F10 melanoma cells were obtained from the KCLB and cultured in DMEM medium supplemented with 10% FBS (Gibco BRL, Grand Island, NY, USA) and 1% penicillin. The cells were incubated in a humidified incubator at 37 °C and 5% CO_2_.

#### 4.7.2. MTT Assay

To determine the cytotoxicity of DP and establish the concentration range for the experiment, an MTT assay was conducted. Cells were seeded in 96-well plates at a density of 1.0 × 10^4^ cells/well. After 24 h, once the cells were fully attached, the medium was replaced with fresh medium containing 200 nM α-MSH and various concentrations of DP. After another 24 h of incubation, an MTT assay was performed.

#### 4.7.3. Measurement of Melanin Content

To measure melanin production in B16F10 cells, cells were seeded in 6-well plates at a density of 5 × 10^4^ cells/well. After 24 h, the medium was replaced with fresh medium containing 2% FBS, 200 nM α-MSH, and various concentrations of DP. The cells were then incubated for 72 h. After incubation, the cells were washed with PBS, harvested by centrifugation, and the melanin content was visually inspected. The harvested cells were dissolved in 400 μL of 0.2 N NaOH solution and incubated at 60 °C for 1 h. The absorbance was measured at 405 nm using a microplate reader.

#### 4.7.4. RT-PCR

Total RNA was extracted using the High Pure RNA Isolation Kit (Invitrogen Co., Waltham, MA, USA) according to the manufacturer’s instructions and quantified using a NanoDrop (Thermo Scientific, Waltham, MA, USA). For the reverse transcription reaction, 1 μg of isolated RNA and 1 μL of oligo DT (100 pM) were combined with DEPC to a total volume of 11 μL, followed by the reverse transcription reaction using the RT Premix Kit (Bioneer Co., Daejeon, Republic of Korea) as per the manufacturer’s protocol. PCR for *MITF*, *Tyrosinase*, and *MC1R* genes was conducted using 2 μL of the cDNA products obtained from the reverse transcription reaction, with the PCR Premix Kit (Bioneer Co.) according to the manufacturer’s instructions. The cDNA products amplified by reverse transcription and polymerase chain reaction were analyzed by electrophoresis on 2.0% agarose gels containing ethidium bromide. The primer sequences used for PCR are provided in Table 1.

### 4.8. Molecular Docking Analysis

#### 4.8.1. Receptor Grid Generation 

For grid generation, the catalytic pocket is selected from its crystallized ligand and literature [48]. The grid was generated by specifying the crystallized ligand of the active site of the target protein. The receptor grid box was defined as 20 Å box. After grid preparation, Glide dock precision docking experiment was performed with default docking setup parameters reporting the top-ranked poses per ligand [49]. The predicted binding scores (binding energies, Supplement S1) and proper orientation of ligands within the catalytic region of tyrosinase were also performed. Finally, the most favorable binding mode of active compounds within the binding pocket was investigated in terms of docking score, and 3D graphical images of the binding pose of the best-docked score were visualized with Maestro (Schrödinger, New York, NY, USA).

#### 4.8.2. Molecular Docking

Molecular docking of mushroom tyrosinase (PDB ID: 2Y9X) was performed by the Ligand Docking module in Schrödinger Suite (Schrödinger software version 2024-1, LLC, New York, NY, USA). The active site of human tyrosinase was similar to that of mushroom tyrosinase (PDB ID: 2Y9X), which contained two metal ions coordinated with six histidine residues [48].

### 4.9. Statistical Analysis

All experimental results are presented as mean ± standard deviation. Statistical significance was verified using ANOVA with the SPSS statistics software (ver. 25, IBM Co., Armonk, NY, USA). The statistical significance of the mean values was analyzed using Duncan’s multiple range test at the *p* < 0.05 level.

## 5. Conclusions

This study evaluates the antioxidant and skin-whitening effects of a novel decapeptide (DP, KGYSSYICDK) derived from marine fish by-products and demonstrates its potential applications. DP exhibits strong antioxidant activity, as evidenced by FRAP and ABTS radical scavenging assays. It effectively enhances cell viability and reduces ROS generation in H_2_O_2_-induced HaCaT cells. Additionally, DP significantly inhibits melanin production in α-MSH-induced B16F10 melanoma cells. This effect is mediated by downregulating the expression of key melanin synthesis-related genes, including *MITF*, *tyrosinase*, and *MC1R*. In silico analysis indicates that DP interacts effectively with the tyrosinase protein, showing substantial inhibitory activity. These findings suggest that DP could be a promising cosmetic ingredient with both antioxidant and skin-whitening properties. Future research should focus on optimizing DP’s structure and exploring its structure–activity relationship to enhance its whitening efficacy.

## Figures and Tables

**Table 1:** Primers used in this study.

Gene	Sequences	
*MITF*	Forward	5′-CCCGTCTCTGGAAACTTGATCG-3′
	Reverse	5′-CTGTACTCTGAGCAGGTG-3′
*Tyrosinase*	Forward	5′-GGCCAGCTTTCAGGCAGAGGT-3′
	Reverse	5′-TGGTGCTTCATGGGCAAAATC-3′
*MC1R*	Forward	5′-CCTCTGCCTCAAGGGTGCTG-3′
	Reverse	5′-TCAACAGTGGAGCTGAGGACG-3′
*GAPDH*	Forward	5′-CCAGTATGACTCCACTCACG-3′
	Reverse	5′-CCTTCCACAATGCCAAGTT-3′

## Data Availability

Data are contained within the article and Supplementary Materials.

## Supplementary Materials

The following supporting information can be downloaded at: https://www.mdpi.com/article/10.3390/md22080374/s1, Supplement S1, binding energies.

## References

[1] Black D.L., Chatterjee R., Hannon D.P. (2008). Chronic ultraviolet radiation-induced increase in skin iron and the photoprotective effect of topically applied iron chelators. Photochem. Photobiol..

[2] Kubo M., Matsuda H. (1995). Development studies of cuticle and medicinal drugs from natural sources on melanin biosynthesis. Fragr. J..

[3] Cadenas D.E. (1989). Biochemistry of oxygen toxicity. Annu. Rev. Biochem..

[4] Kwon M.H., Choi S.Y., Kim Y.C. (2009). Inhibitory Effects of Peonia japonica Water Extract on Skin Aging (II) -Focused on Inhibitory Effects of Wrinkle Formation. J. Environ. Toxicol..

[5] Ku B., Kim D., Choi E. (2019). Tetrahydrocurcumin Inhibits α-MSH-induced Melanogenesis via GSK3β Activation in B16F10 Melanoma Cells. Toxicol. Environ. Health Sci..

[6] Ku B., Kim D., Choi E. (2020). Anti-melanogenic effect of the aqueous ethanol extract of *Ginkgo biloba* leaf in B16F10 cells. Toxicol. Environ. Health Sci..

[7] Ye Y., Chou G.X., Wang H., Chu J.H., Yu Z.L. (2010). Flavonoids, apigenin and icariin exert potent melanogenic activities in murine B16 melanoma cells. Phytomedicine.

[8] Yamakoshi J., Otsuka F., Sano A., Tokutake S., Saito M., Kikuchi M., Kubota Y. (2003). Lightening effect on ultraviolet-induced pigmentation of Guinea pig skin by oral administration of a proanthocyanidin-rich extract from grape seeds. Pigment Cell Res..

[9] Pillaiyar T., Manickam M., Namasivayam V. (2017). Skin whitening agents: Medicinal chemistry perspective of tyrosinase inhibitors. J. Enzym. Inhib. Med. Chem..

[10] Juhasz M.L.W., Levin M.K. (2018). The role of systemic treatments for skin lightening. J. Cosmet. Dermatol..

[11] Wu H.C., Chen H.M., Shiau C.Y. (2003). Free amino acids and peptides as related to antioxidant properties in protein hydrolysates of mackerel (*Scomber austriasicus*). Food Res. Int..

[12] Kim S.K. (2013). Marine Proteins and Peptides: Biological Activities and Applications.

[13] Lintner K., Peschard O. (2000). Biologically active peptides: From a laboratory bench curiosity to a functional skin care product. Int. J. Cosmet. Sci..

[14] Khora S.S. (2013). Marine fish-derived bioactive peptides and proteins for human therapeutics. Int. J. Pharm. Pharm. Sci..

[15] Halim N., Yusof H., Sarbon N. (2016). Functional and bioactive properties of fish protein hydrolysates and peptides: A comprehensive review. Trends Food Sci. Technol..

[16] Sila A., Hedhili K., Przybylski R., Ellouz-Chaabouni S., Dhulster P., Bougatef A., Nedjar-Arroume N. (2014). Antibacterial activity of new peptides from barbel protein hydrolysates and mode of action via a membrane damage mechanism against Listeria monocytogenes. J. Funct. Foods.

[17] Hwang J.W., Lee S.G., Kang H. (2024). Antioxidant, Antibacterial Properties of Novel Peptide CP by Enzymatic Hydrolysis of *Chromis notata* By-Products and Its Efficacy on Atopic Dermatitis. Mar. Drugs.

[18] Senevirathne M., Kim S.K., Kim S.K. (2012). Utilization of seafood processing by-products: Medicinal applications. Advances in Food and Nutrition Research.

[19] Rustad T., Shahidi F. (2007). Physical and chemical properties of protein seafood by-products. Maximising the Value of Marine By-Products.

[20] Nilsang S., Lertsiri S., Suphantharika M., Assavanig A. (2005). Optimization of enzymatic hydrolysis of fish soluble concentrate by commercial proteases. J. Food Eng..

[21] Shahidi F., Kamil Y.J. (2001). Enzymes from fish and aquatic invertebrates and their application in the food industry. Trends Food Sci. Technol..

[22] Hoyer B., Bernhardt A., Heinemann S., Stachel I., Meyer M., Gelinsky M. (2012). Biomimetically mineralized salmon collagen scaffolds for application in bone tissue engineering. Biomacromolecules.

[23] Lauritano C., Ianora A. (2016). Marine organisms with anti-diabetes properties. Mar. Drugs.

[24] Schagen S.K. (2017). Topical peptide treatments with effective anti-aging results. Cosmetics.

[25] Kim S.K., Wijesekara I. (2010). Development and biological activities of marine-derived bioactive peptides: A review. J. Funct. Foods.

[26] Ngo D.H., Vo T.S., Ryu B., Kim S.K. (2012). Biological activities and potential health benefits of bioactive peptides derived from marine organisms. Int. J. Biol. Macromol..

[27] Iwaniak A., Darewicz M., Minkiewicz P. (2014). Peptides derived from food proteins as natural antioxidants. Curr. Opin. Food Sci..

[28] Marathe S.A., Rajalakshmi V., Jamdar S.N., Sharma A. (2011). Comparative study on antioxidant activity of different varieties of commonly consumed legumes in India. Food Chem. Toxicol..

[29] Reczek C.R., Chandel N.S. (2015). ROS-dependent signal transduction. Curr. Opin. Cell Biol..

[30] Pessetto Z.Y., Weir S.J., Sethi G., Broward M.A., Godwin A.K. (2013). Drug repurposing for gastrointestinal stromal tumor. Mol. Cancer Ther..

[31] Han J.S., Sung J.H., Lee S.K. (2016). Antimelanogenesis activity of hydrolyzed ginseng extract (GINST) via inhibition of JNK mitogen-activated protein kinase in B16F10 cells. J. Food Sci..

[32] Chatatikun M., Chiabchalard A. (2017). Thai plants with high antioxidant levels, free radical scavenging activity, anti-tyrosinase and anti-collagenase activity. BMC Complement. Altern. Med..

[33] Garcia-Jimenez A., Teruel-Puche J.A., Berna J., Rodriguez-Lopez J.N., Tudela J., Garcia-Canovas F. (2017). Action of tyrosinase on alpha and beta-arbutin: A kinetic study. PLoS ONE.

[34] Yu J.S., Kim A.K. (2010). Effect of combination of taurine and azelaic acid on antimelanogenesis in murine melanoma cells. J. Biomed. Sci..

[35] Lee C.S., Jang W.H., Park M., Jung K., Baek H.S., Joo Y.H., Park Y.H., Lim K.M. (2013). A novel adamantyl benzylbenzamide derivative, AP736, suppresses melanogenesis through the inhibition of cAMP-PKA-CREB-activated microphthalmia-associated transcription factor and tyrosinase expression. Exp. Dermatol..

[36] Chang H., Choi H., Joo K.M., Kim D., Lee T.R. (2012). Manassantin B inhibits melanosome transport in melanocytes by disrupting the melanophilin-myosin Va interaction. Pigment Cell Melanoma Res..

[37] Makino-Okamura C., Niki Y., Takeuchi S., Nishigori C., Declercq L., Yaroch D.B., Saito N. (2014). Heparin inhibits melanosome uptake and inflammatory response coupled with phagocytosis through blocking PI3k/Akt and MEK/ERK signaling pathways in human epidermal keratinocytes. Pigment Cell Melanoma Res..

[38] Seiberg M., Paine C., Sharlow E., Andrade-Gordon P., Costanzo M., Eisinger M., Shapiro S.S. (2000). The protease-activated receptor 2 regulates pigmentation via keratinocyte-melanocyte interactions. Exp. Cell Res..

[39] Kippenberger S., Loitsch S., Solano F., Bernd A., Kaufmann R. (1998). Quantification of tyrosinase, TRP-1, and Trp-2 transcripts in human melanocytes by reverse transcriptase-competitive multiplex PCR–regulation by steroid hormones. J. Investig. Dermatol..

[40] Park Y.D., Kim S.Y., Lyou Y.J., Lee D.Y., Yang J.M. (2006). TXM13 human melanoma cells: A novel source for the inhibition kinetics of human tyrosinase and for screening whitening agents. Biochem. Cell Biol..

[41] Tuerxuntayi A., Liu Y.Q., Tulake A., Kabas M., Eblimit A., Aisa H.A. (2014). Kaliziri extract upregulates tyrosinase, TRP-1, TRP-2 and MITF expression in murine B16 melanoma cells. BMC Complement. Altern. Med..

[42] Levy C., Khaled M., Fisher D.E. (2006). MITF: Master regulator of melanocyte development and melanoma oncogene. Trends Mol. Med..

[43] Tu M., Cheng S., Lu W., Du M. (2018). Advancement and prospects of bioinformatics analysis for studying bioactive peptides from food-derived protein: Sequence, structure, and functions. Trends Anal. Chem..

[44] Zhu D., Huang H., Pinkas D.M., Luo J., Ganguly D., Fox A.E., Arner E., Xiang Q., Tu Z.C., Bullock A.N. (2019). 2-Amino-2,3-dihydro-1H-indene-5-carboxamide-based discoidin domain receptor 1 (DDR1) inhibitors: Design, synthesis, and in vivo antipancreatic cancer efficacy. J. Med. Chem..

[45] Re R., Pellegrini N., Proteggente A., Pannala A., Yang M., Rice-Evans C. (1999). Antioxidant activity applying an improved ABTS radical cation decolorization assay. Free Radic. Biol. Med..

[46] Benzie I.F., Strain J.J. (1996). The ferric reducing ability of plasma (FRAP) as a measure of antioxidant power the FRAP assay. Anal. Biochem..

[47] Masamoto Y., Ando H., Murata Y., Shimoishi Y., Tada M., Takahata K. (2003). Mushroom tyrosinase inhibitory activity of esculetin isolated from seeds of *Euphorbia lathyris* L.. Biosci. Biotechnol. Biochem..

[48] Ismaya W.T., Rozeboom H.J., Weijn A., Mes J.J., Fusetti F., Wichers H.J., Dijkstra B.W. (2011). Crystal Structure of Agaricus bisporus Mushroom Tyrosinase: Identity of the Tetramer Subunits and Interaction with Tropolone. Biochemistry.

[49] Friesner R.A., Murphy R.B., Repasky M.P., Frye L.L., Greenwood J.R., Halgren T.A., Sanschagrin P.C., Mainz D.T. (2006). Extra Precision Glide: Docking and Scoring Incorporating a Model of Hydrophobic Enclosure for Protein–Ligand Complexes. J. Med. Chem..

