# Supplementary material for: Antioxidant and Skin-Whitening Efficacy of a Novel Decapeptide (DP, KGYSSYICDK) Derived from Fish By-Products
Source: Mar Drugs. 2024 Aug 20;22(8):374. doi: 10.3390/md22080374 (PMC11355700; doi:10.3390/md22080374)

Supplement S1, binding energies

Binding score

(kcal/mol)

2Y9X		Docking score	MMGBSA score
**Control**	**KVARP**	**-6.66**	**-18.40**
**Ligand01**	**KGYSSYICDK**	**-8.64**	**-19.35**
**Ligand02**	**L-TYROSINE**	**-9.01**	**-14.60**
**Ligand03**	**L-DOPA**	**-8.39**	**-15.08**

Ligand 2D

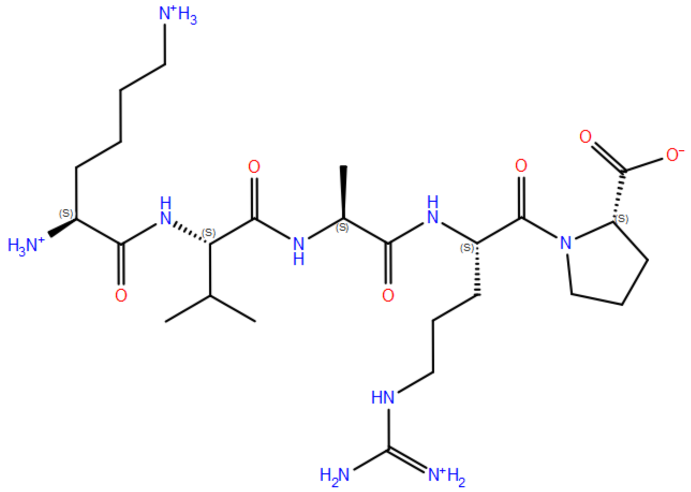

Control: KVARP

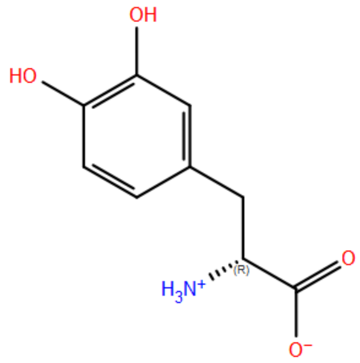

Ligand02: L-Tyrosine

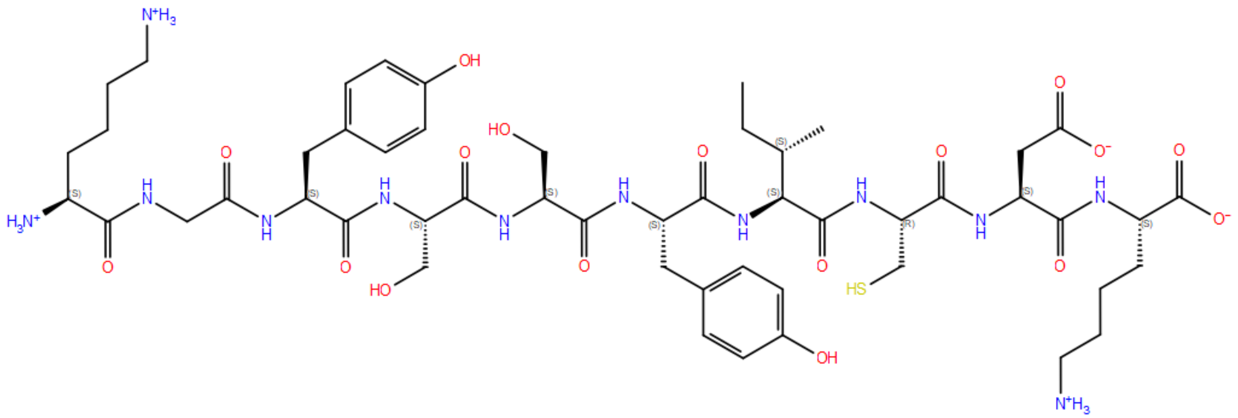

Ligand01: KGYSSYICDK

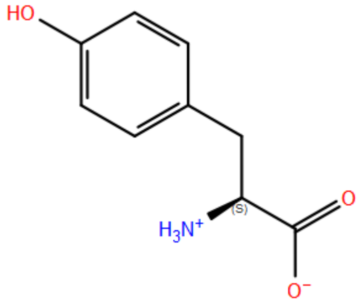

Ligand03: L-DOPA

Ligand 3D

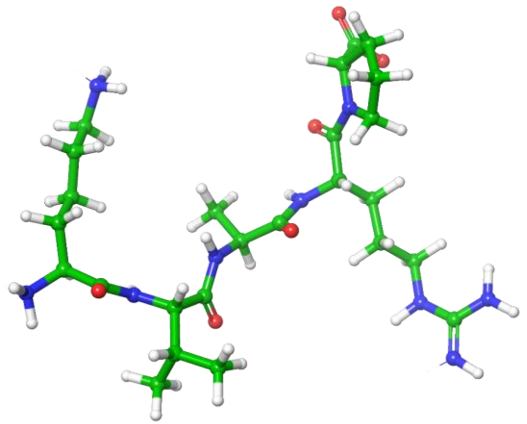

Control: KVARP

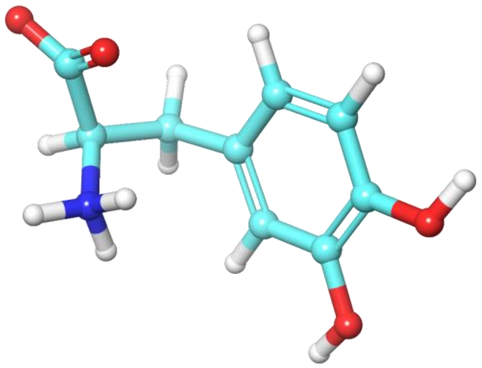

Ligand02: L-Tyrosine

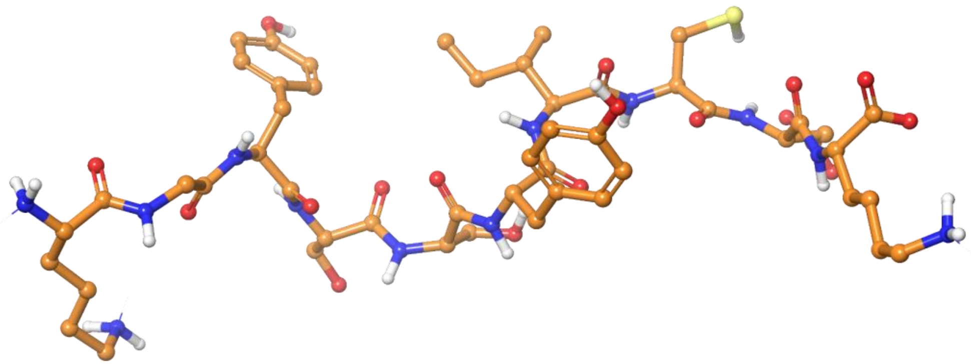

Ligand01: KGYSSYICDK

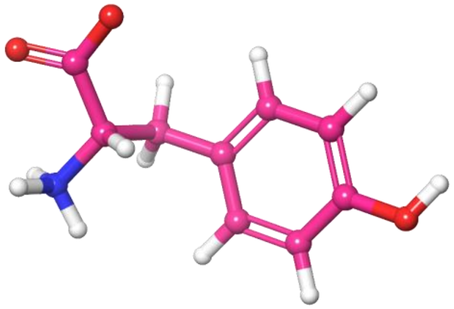

Ligand03: L-DOPA

Tyrosinase PDB

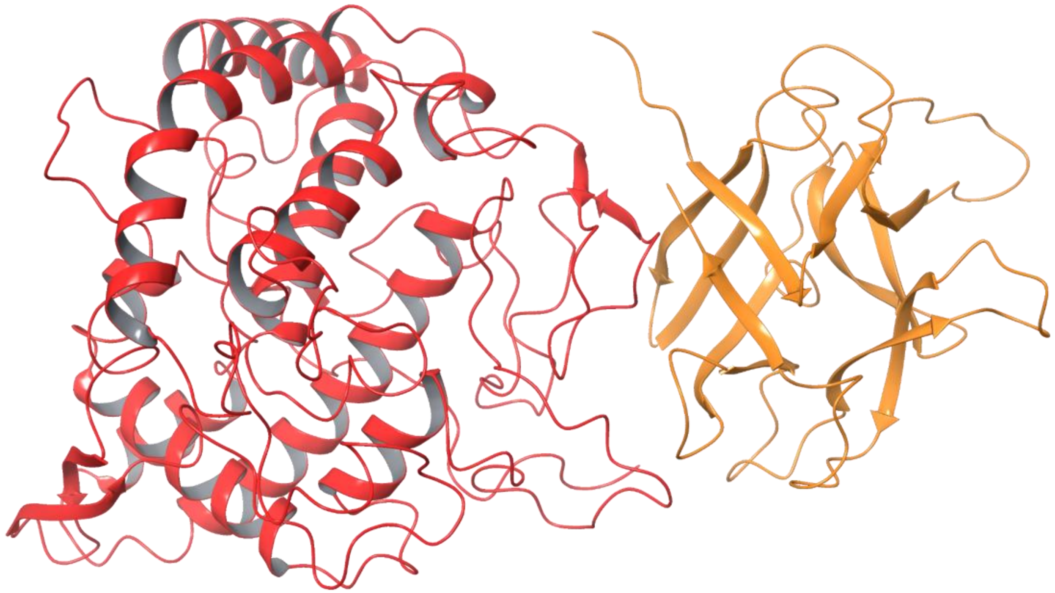

PDB 2Y9X

Grid box in this experiment

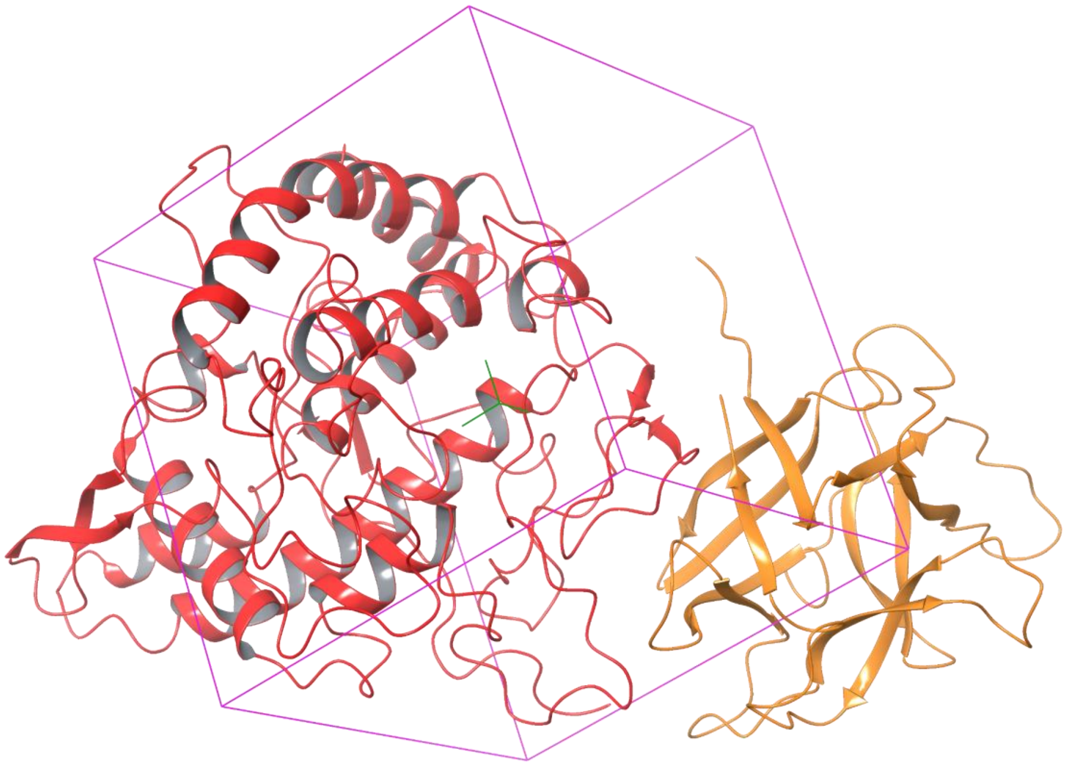

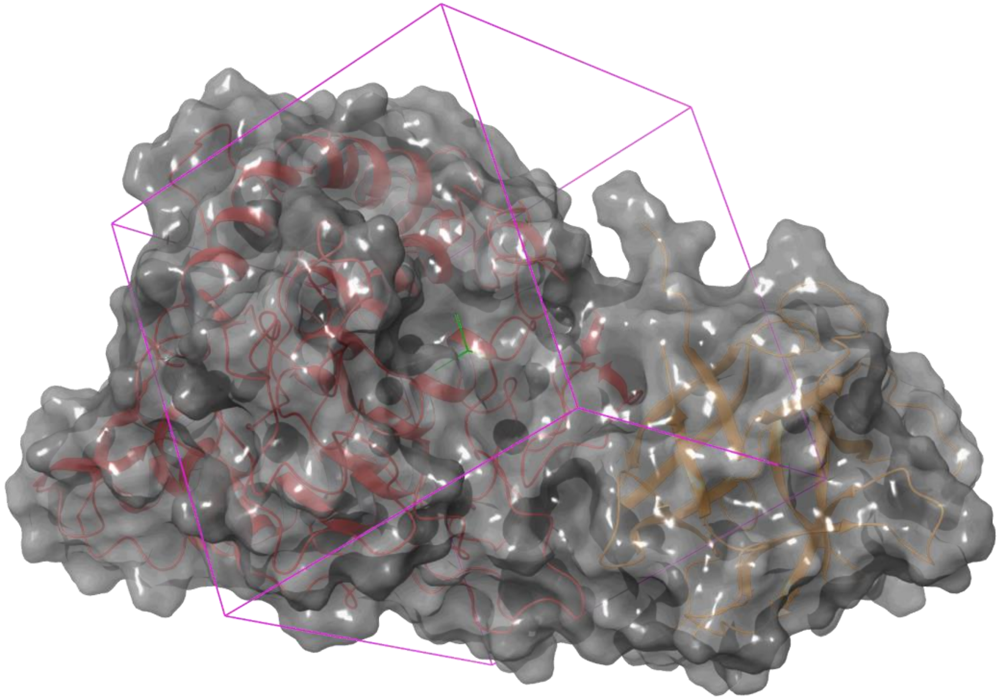

Docking result – Ligand01

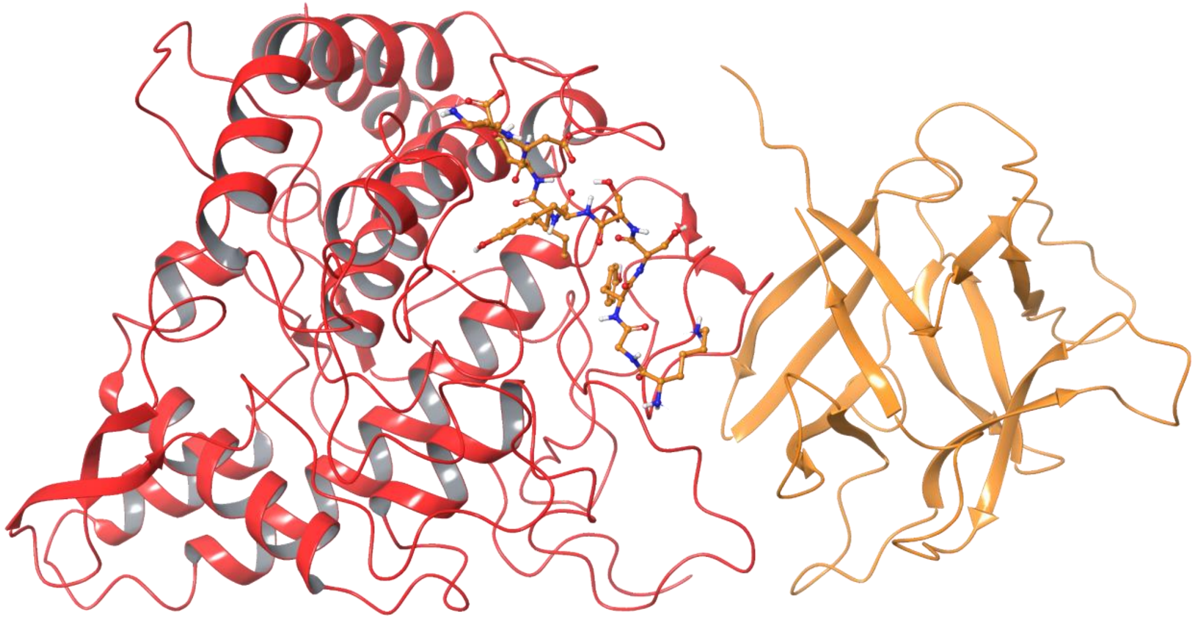

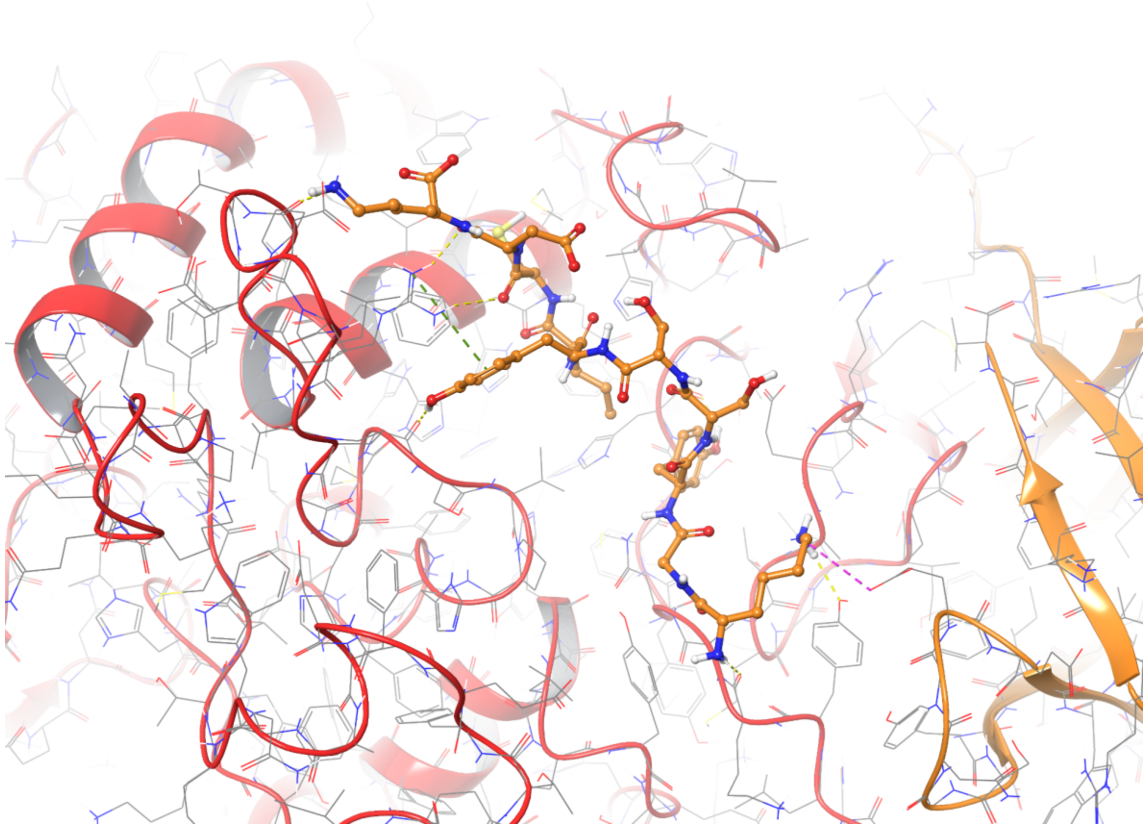

Docking result – Ligand02

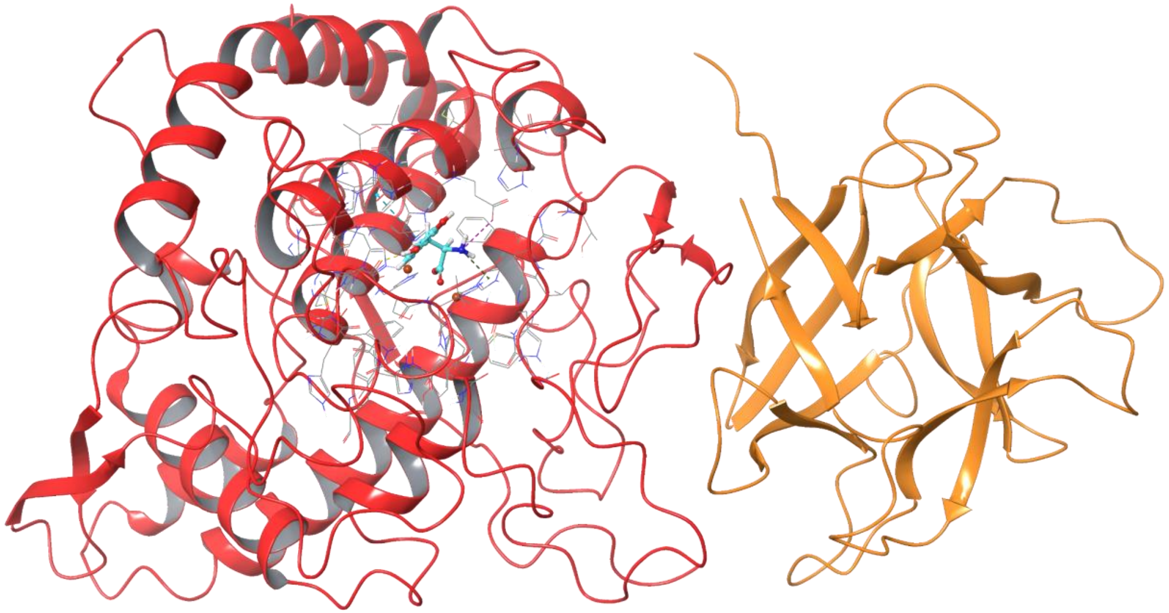

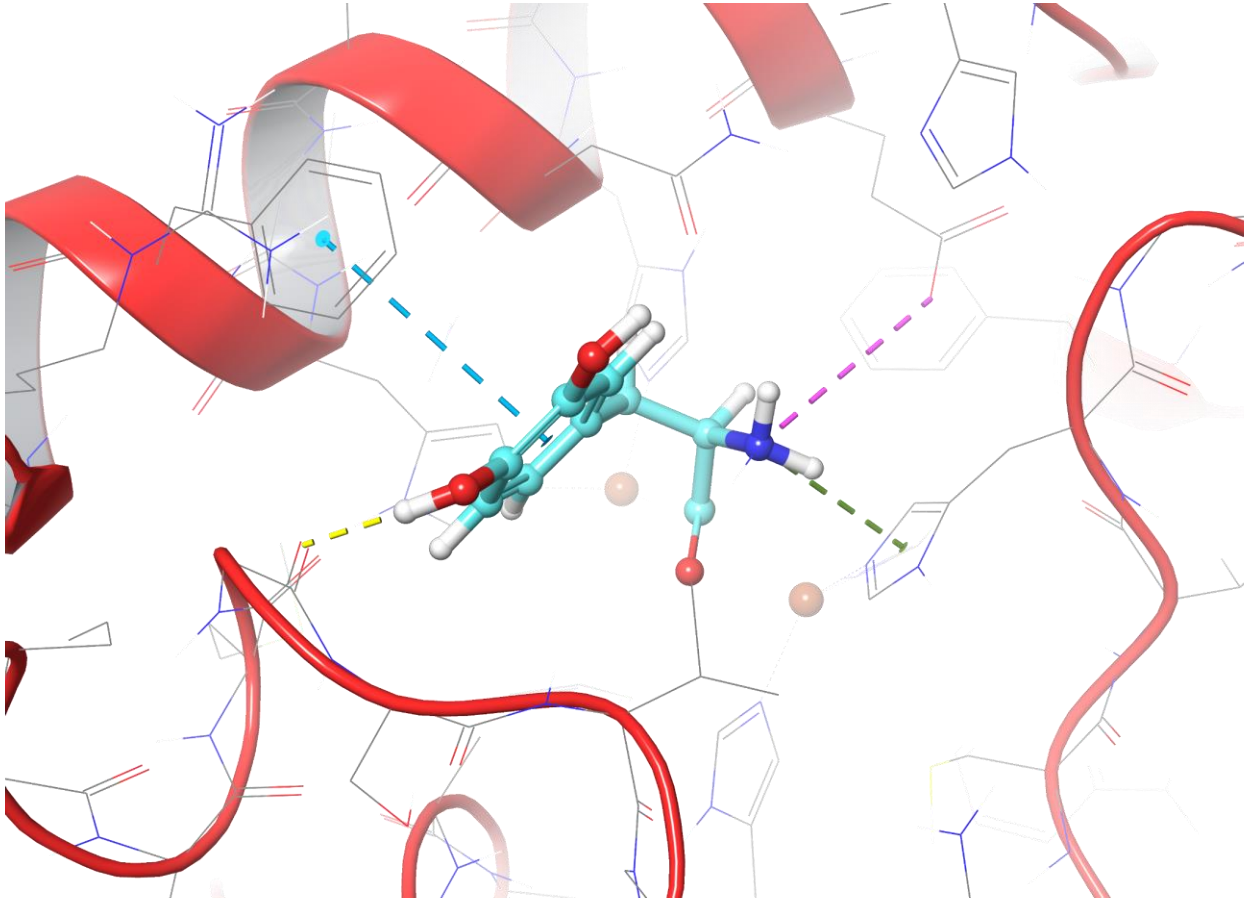

Docking result – Ligand03

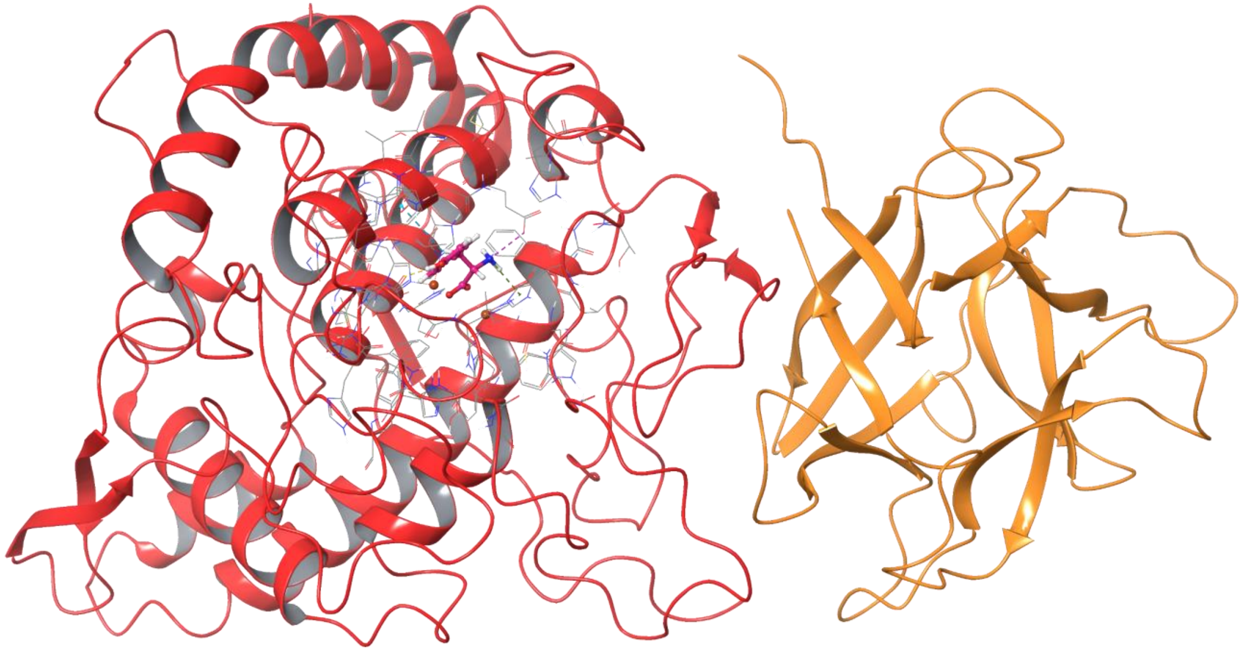

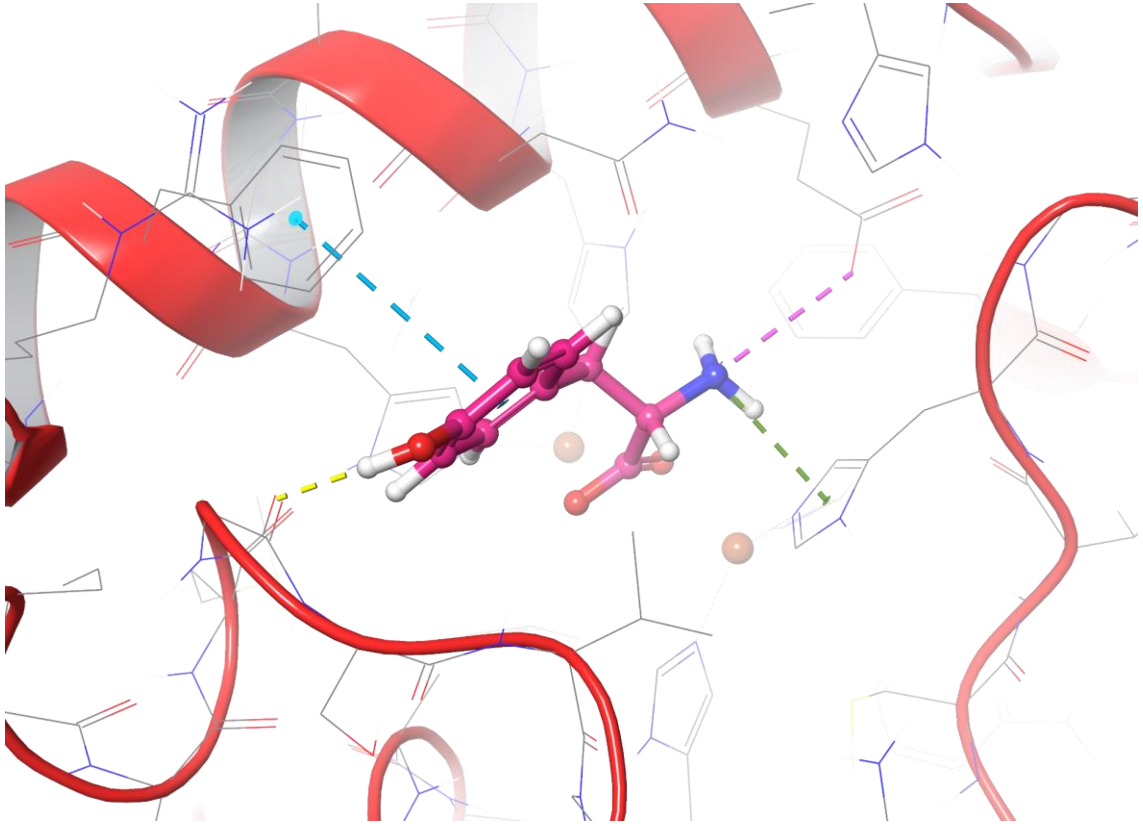

Docking result – Control (2D)

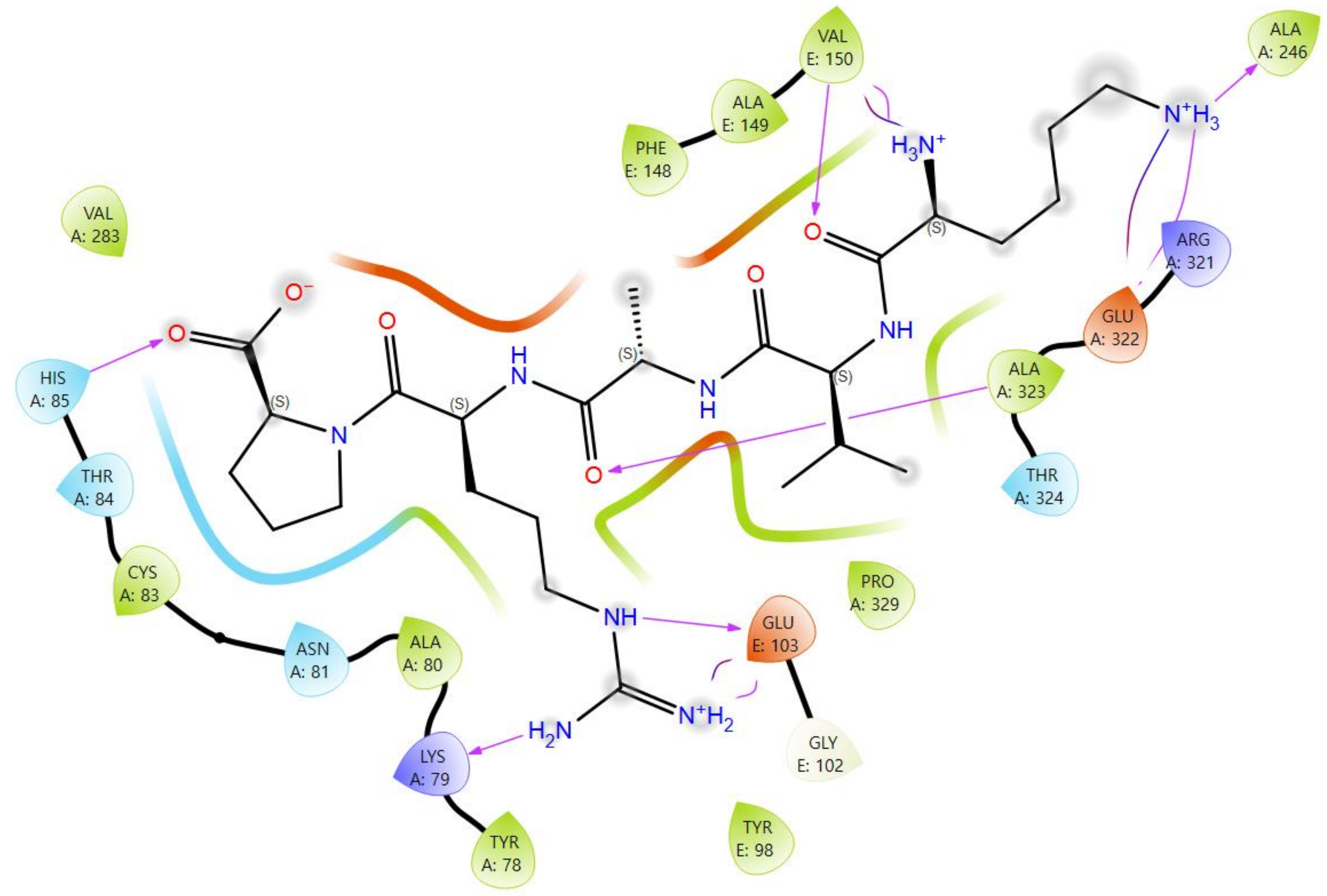

Docking result – Ligand01 (2D)

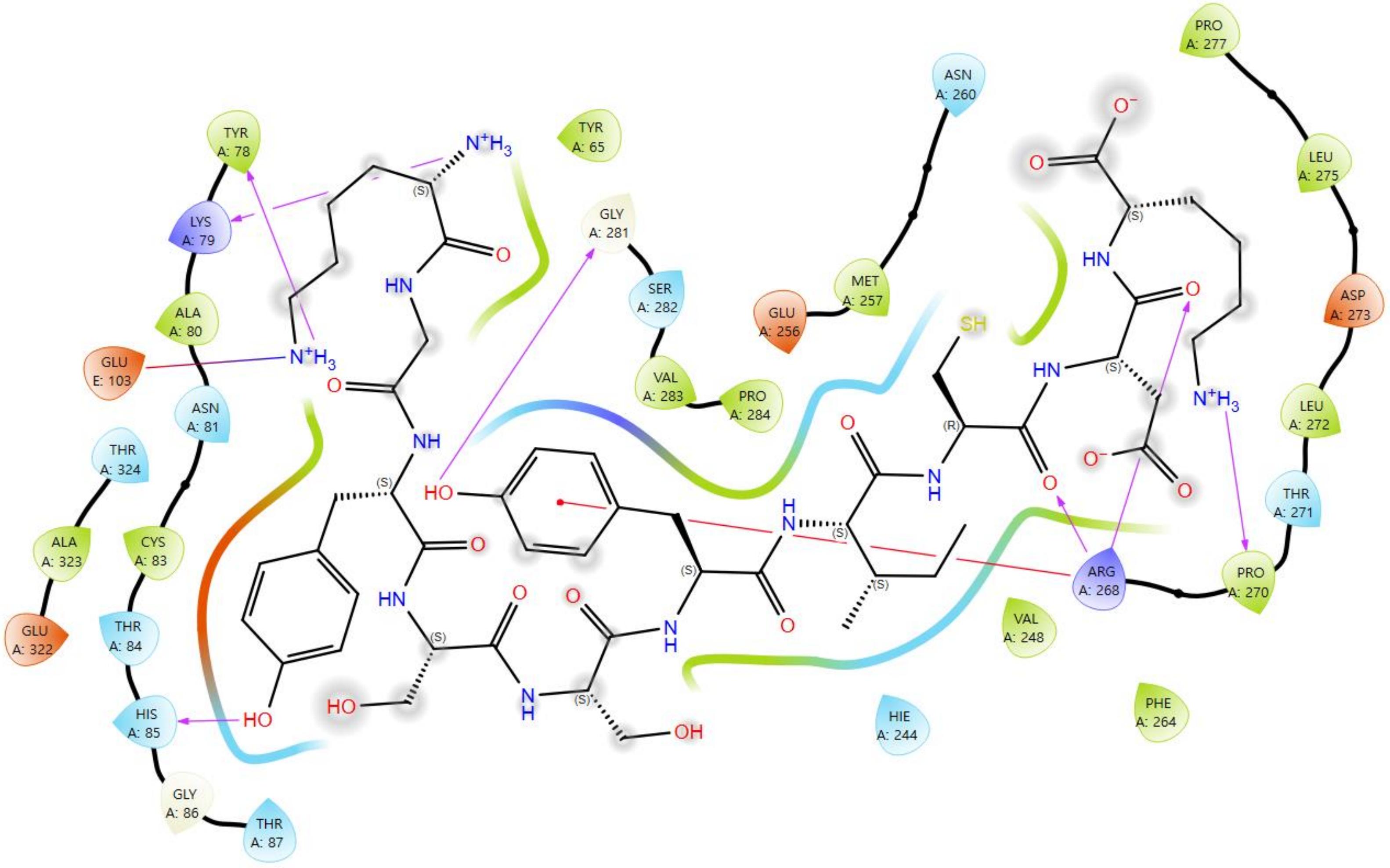

Docking result – Ligand02 (2D)

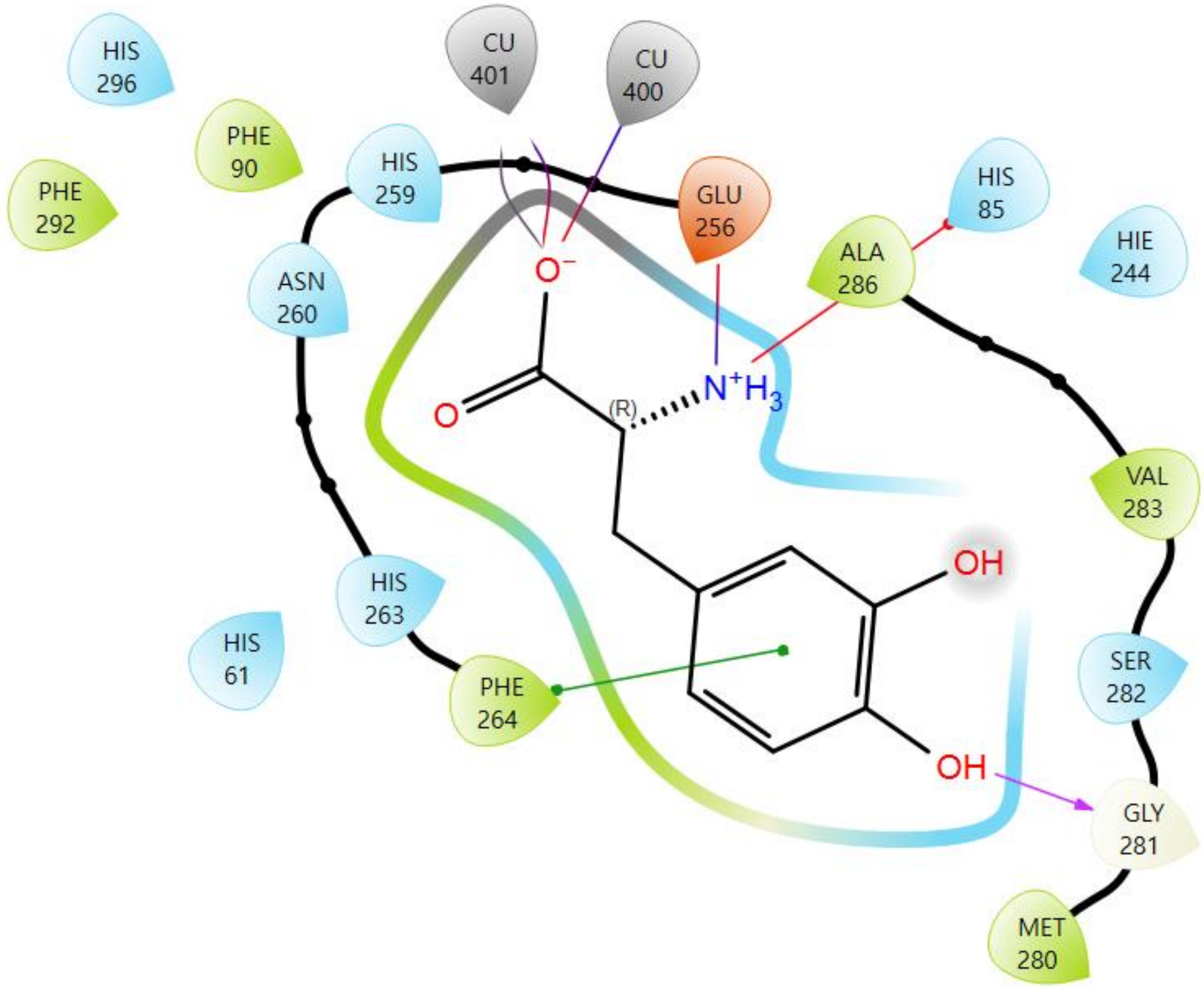

Docking result – Ligand03 (2D)

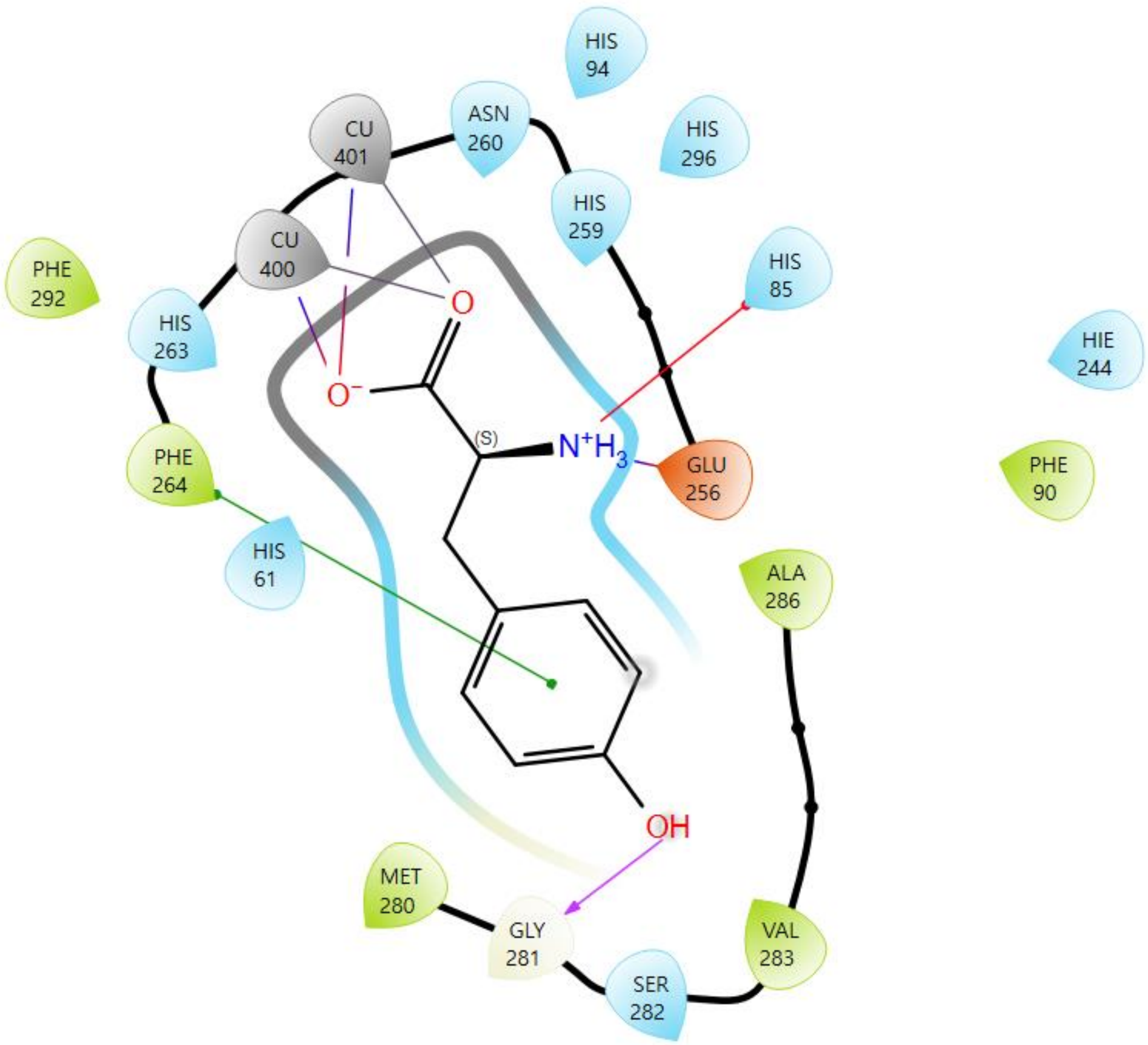

Docking result – Control (binding site)

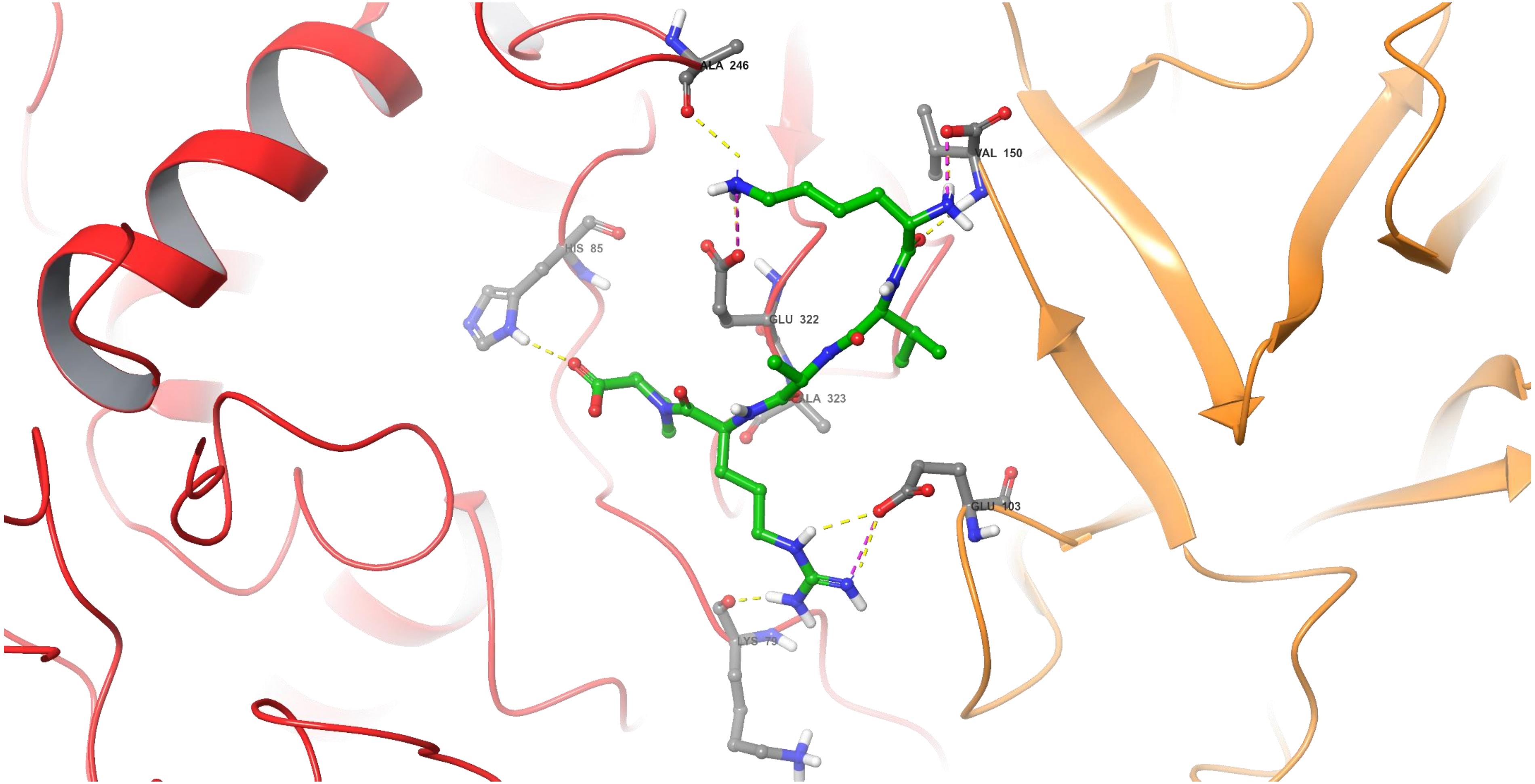

Docking result – Ligand01 (binding site)

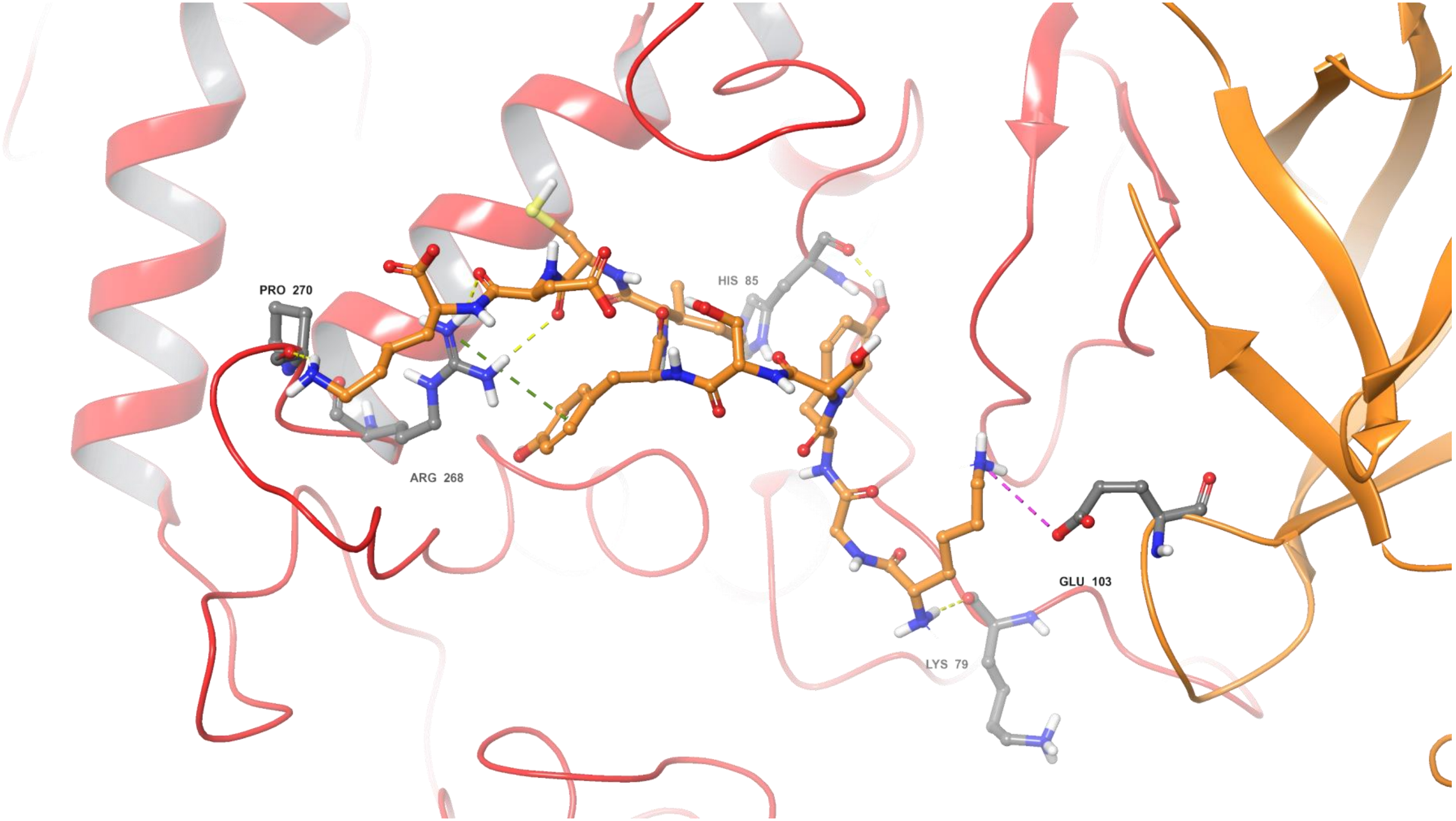

Docking result – Ligand02 (binding site)

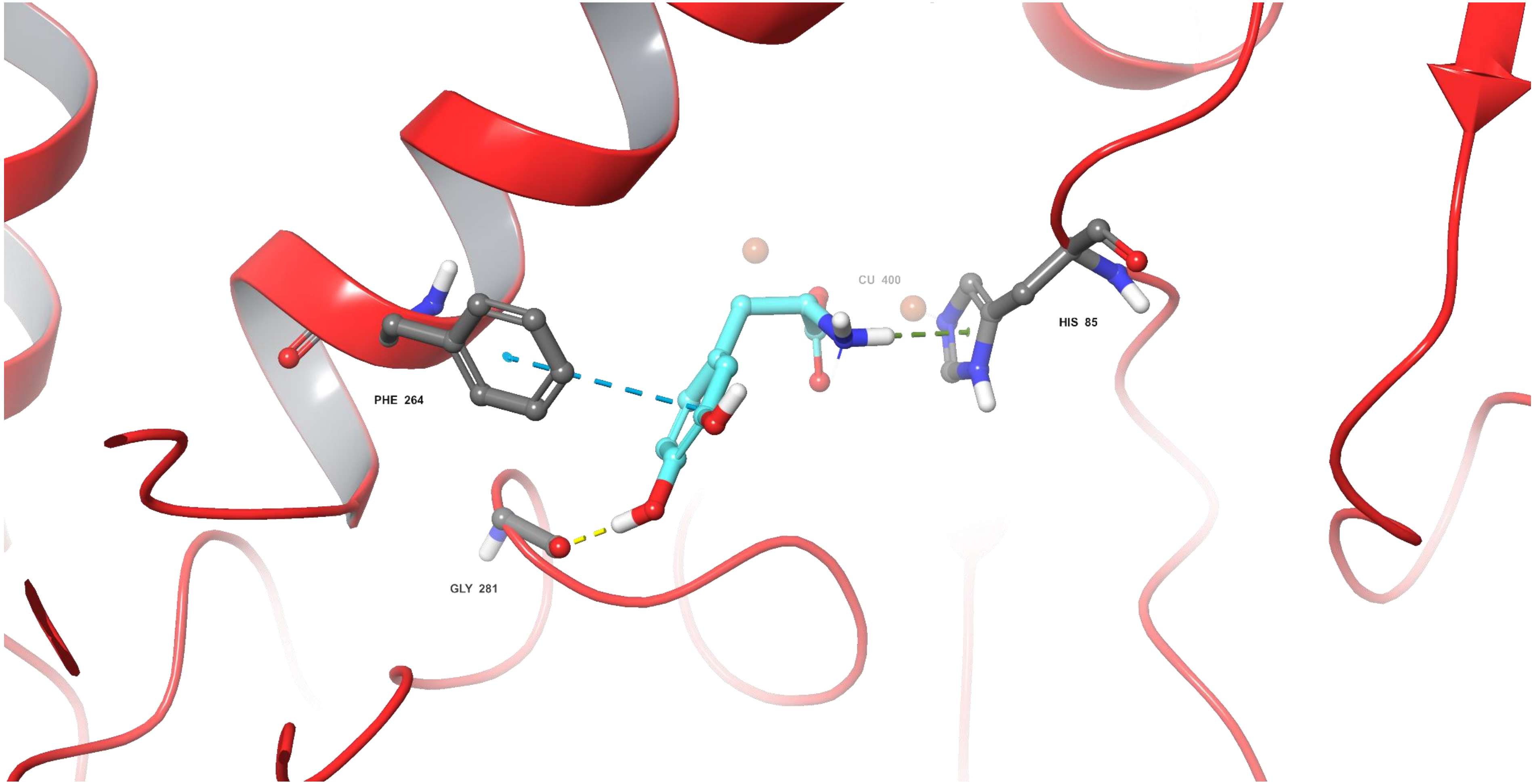

Docking result – Ligand03 (binding site)

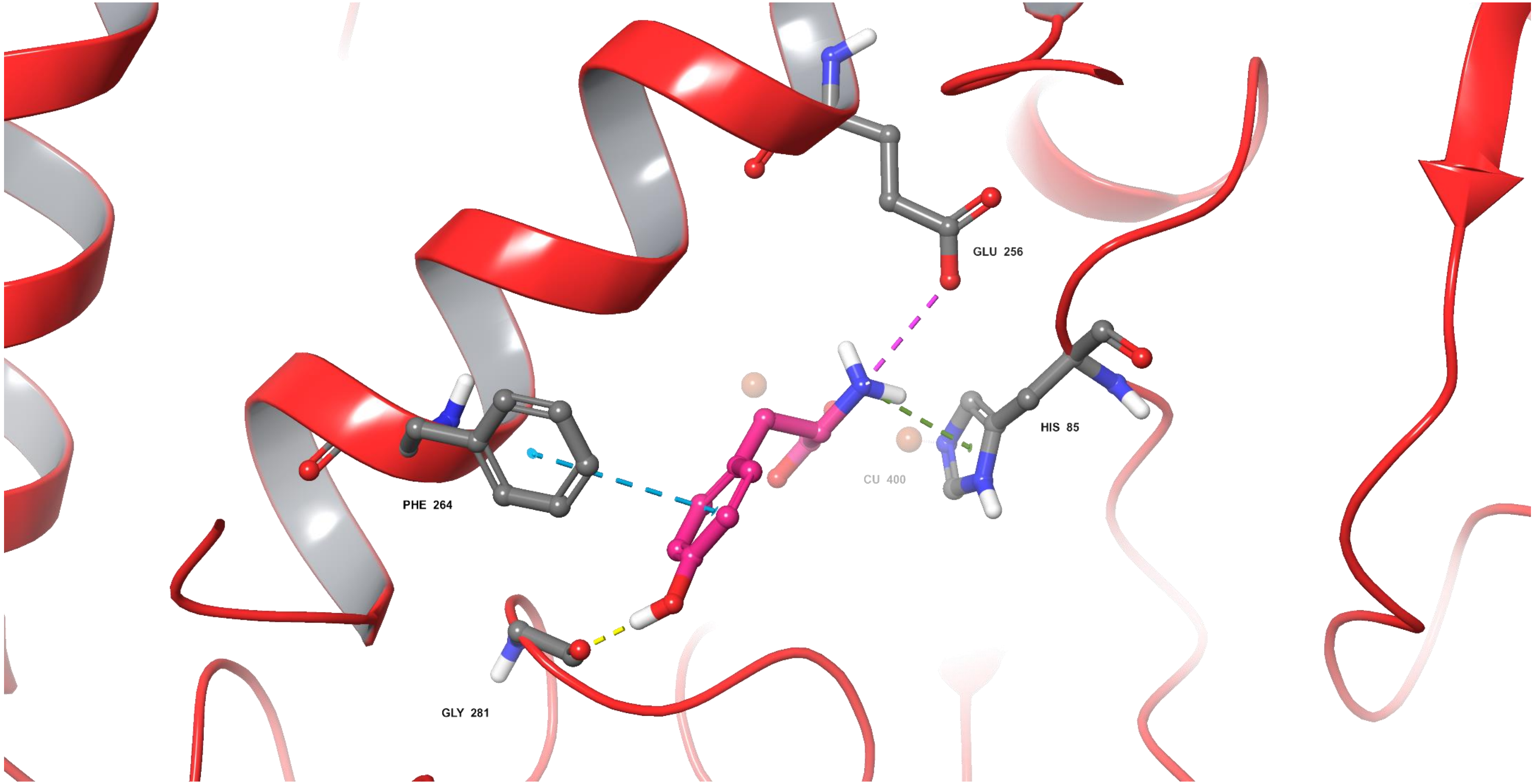

Supplement: Supplementary file 1 [file marinedrugs-22-00374-s001.zip › marinedrugs-3140984-supplementary.pdf]
